# Coffee Silverskin Phytocompounds as a Novel Anti-Aging Functional Food: A Pharmacoinformatic Approach Combined with In Vitro Study

**DOI:** 10.3390/molecules28207037

**Published:** 2023-10-11

**Authors:** Clarin Hayes, Fahrul Nurkolis, Dewa Ayu Agus Sri Laksemi, Sanghyun Chung, Moon Nyeo Park, Min Choi, Jinwon Choi, I Gusti Nyoman Darmaputra, William Ben Gunawan, Juan Alessandro Jeremis Maruli Nura Lele, Mohammad Adib Khumaidi, Nurpudji Astuti Taslim, Bonglee Kim

**Affiliations:** 1 Faculty of Medicine, Udayana University, Denpasar 80361, Indonesia; 2 Department of Biological Sciences, State Islamic University of Sunan Kalijaga (UIN Sunan Kalijaga), Yogyakarta 55281, Indonesia; fahrul.nurkolis.mail@gmail.com; 3 Department of Pathology, College of Korean Medicine, Kyung Hee University, Hoegidong Dongdaemun-gu, Seoul 02447, Republic of Korea; 4 Kyung Hee Myungbo Clinic of Korean Medicine, Hwaseong-si 18466, Republic of Korea; 5 Korean Medicine-Based Drug Repositioning Cancer Research Center, College of Korean Medicine, Kyung Hee University, Seoul 02447, Republic of Korea; 6 Department of Nutrition Science, Faculty of Medicine, Diponegoro University, Semarang 50275, Indonesia; wbwilliambenwb@gmail.com; 7 Faculty of Medicine, Universitas Kristen Indonesia, Jakarta 13630, Indonesia; juanalessandro.uki@gmail.com; 8 Faculty of Medicine and Health, Universitas Muhammadiyah Jakarta, Jakarta 15419, Indonesia; 9 Division of Clinical Nutrition, Department of Nutrition, Faculty of Medicine, Hasanuddin University, Makassar 90245, Indonesia

**Keywords:** coffee silverskin extract, functional food, anti-aging, antioxidant capabilities, molecular docking, coffee silverskin phytocompounds, catechin, epicatechin, kaempferol, quercitrin, naringin

## Abstract

Coffee became a beverage that was in demand in the world and consequently produced millions of tons of coffee byproducts namely coffee silverskin (CS). Unutilized CS will be waste and cause environmental pollution such as greenhouse gas emissions, landfill waste, and groundwater contamination. This is a research concern at this time, although many studies have been conducted to find newer applications of CS, exploration of its benefits in the health sector is still limited. Therefore, exploring the benefits of CS to prevent or delay aging will be very interesting to develop in functional food industry technology. Therefore, this study aims to report profiling metabolites or phytochemicals, biological activities in terms of antioxidant activity, and potential anti-aging of CS via molecular docking simulation and in vitro modulation of the mTOR/AMPK/SIRT1 pathway. Something new has been obtained from this work, the profile of phytocompounds, and biological activities both in molecular docking simulation and in vitro studies. Some of the compounds observed in Robusta CS extract (rCSE) such as Epicatechin, Kaempferol, and Quercitrin, and Arabica CS extract (aCSE) such as (+)-Catechin dan Naringin have promising potential as inhibitors of iNOS, mTOR, and HIF-1α via molecular docking simulation. Interestingly, the in vitro biological activity assay of antioxidant and anti-aging activity, rCSE showed the same promising potential as the results of a molecular docking simulation. More interestingly, AMPK/SIRT1/mTOR expressions are well modulated by rCSE compared to aCSE significantly (*p* < 0.05). This makes the rCSE have promising biological activity as a candidate for functional food development and/or treatment agent in combating free radicals that cause the aging process. In vivo studies and human trials are certainly needed to see the further efficacy of the rCSE in the future.

## 1. Introduction

Fundamentally, the process of skin aging comprises the cumulative effects of both intrinsic biological aging and extrinsic aging instigated by external environmental elements, including pollution, exposure to cigarette smoke, ultraviolet (UV) radiation, and inflammatory stimuli [1], in addition to the possibility of aging owing to climate change [2]. The aging process is characterized by the formation of reactive oxygen species (ROS) via signal transduction, activation of mTOR/AP-1 signaling proteins, and downregulation of SIRT-1/AMPK signaling [3]. The anti-aging discovery strategy is interested in observing the modulation of the mTOR/AMPK/SIRT1 pathway as a result of an intervention [4].

Aging is a unidirectional physiological phenomenon, but unhealthy consequences can be avoided through a healthy diet. Functional foods and ‘nutraceuticals’ encompass all kinds of foods with health or medical effects, a growing trend and area of research interest in the search for anti-aging agents [5]. In accordance with international surveys, up to fifty percent of adults (and presumably one-third of children) consume vitamins, minerals, and dietary supplements daily [6]. This indicates a growing interest in food-derived anti-aging substances, particularly natural ingredients. Since the aging process involves the formation of reactive oxygen species, natural ingredient-based supplements with primarily antioxidant properties are the most popular [7]. This category comprises plants with bioactive secondary metabolites, such as polyphenols, vitamins, prebiotics, isoflavones, phytoestrogens, and omega-3 fatty acids. In addition, hyaluronic acid and collagen peptides, which are the skin’s construction elements, have been marketed [8]. However, there are still a vast number of unexplored natural substances that have the potential to become new anti-aging agents. Coffee silverskin (CS) is a natural ingredient that is also a byproduct of natural ingredients with unexplored anti-aging properties.

Annually, millions of tons of coffee byproduct CS are produced [9]. CS is the thin layer that covers coffee beans as a result of the roasting process [10]. In light of the increase in coffee production and the environmental impact of refuse accumulation, CS disposal must be effectively managed. In recent years, numerous studies have focused on discovering new applications for CS, particularly its health benefits [11,12,13]. Various practical studies on the utilization of CS have emerged, such as its application as a feedstock for biofuel production [14], A material that eliminates the possibility of toxic metals in water [15], and as a raw material for the recovery of potentially interesting functional compounds, such as soluble and water-insoluble fibers [16]. In addition, recent research has demonstrated that CS is a rich source of bioactive compounds, such as polyphenols, which opens the door to the use of CS extract as a functional constituent in healthcare, though this potential has yet to be fully explored [17]. In the latest study, a total of 30 bioactive compounds were also quantified and accompanied by a promising evaluation of their antioxidant and antibacterial activities [16]. However, despite expanding interest in CS, bioactive compounds in CS have not been thoroughly characterized, let alone molecular pathways using pharmacoinformatics or molecular docking approaches. Seeing its bioactive potential, therefore it is very important to analyze the molecular benefits of CS through pharmacoinformatics or molecular docking approaches to see the direct effect of each observed compound on receptors, especially aging receptors which have been discussed in the previous paragraph. In addition, the activity of free radical scavenging through the ABTS (2,2′-azino-bis(3-ethylbenzothiazoline-6-sulfonic acid)) inhibitory approach of CS is still small. 

Indeed, research into value-added applications for CS necessitates additional in-depth investigation to determine the potential of bioactive compounds and their biological activity. No studies have been able to successfully report and combine the pharmacoinformatics approach or molecular docking simulation from CS as anti-aging functional foods until now. In addition, understanding the fundamentals of how each observed compound of CS functions will provide new insight into its molecular benefits and elevate the importance of current research. Consequently, the purpose of this study is to report profiling metabolites, antioxidant activity, and potential anti-aging of CS by pharmacoinformatics and advanced in vitro assays of mTOR/AMPK/SIRT pathway modulation. In addition, biological activity against free radicals was compared between Robusta-coffee silverskin extract (rCSE) and Arabica-coffee silverskin extract (aCSE). This will be a comprehensive new insight that fills the previous knowledge void regarding the benefits of CS as a functional food candidate for anti-aging and its accelerated development potential.

## 2. Results

### 2.1. Metabolites Profile of Two Coffee Silverskin Extract (CSE)

Through HPLC-ESI-HRMS/MS analysis, coffee silverskin (CS), Robusta-coffee silverskin extract (rCSE), and Arabica-coffee silverskin extract (aCSE) were successfully observed and summarized in Table 1. Within the 99.0% best match with the library of mzCloud, each CS was observed with the same number of compounds, with a total of five compounds (Table 1).

In the compound data presented in Table 1, it appears that CS is dominated by flavonoids and followed by several polyphenols. Observed compounds in Table 1 continued to be docked using the molecular docking simulation process on selected aging-related ROS or oxidant receptors, including hypoxia-inducible factor-1 alpha (HIF-1α), human mammalian target of rapamycin (mTOR), human inducible nitric oxide synthase (iNOS) and reactive oxygen species 1 kinase (ROS1).

### 2.2. Pharmacoinformatics via Molecular Docking Simulation of Observed Compounds in Coffee Silverskin Extract

As evidenced in Table 2, the receptors specifically selected for molecular binding assessments, encompassing iNOS, ROS1 Kinase, mTOR, and HIF-1, underwent a rigorous validation process through redocking analysis, which resulted in root mean square deviation (RMSD) values of 2 or lower.

The data analysis summarized in Table 3 from molecular docking simulations demonstrated that S-ibuprofen, the control substance in this case, impacted the inhibition of iNOS. On the other hand, quercetin, genistein, and luteolin exhibited inhibitory effects against mTOR rapamycin, HIF-1, and ROS1. Table 3 outlines the bioactive elements present in rCSE and aCSE that hinder iNOS, mTOR rapamycin, and HIF-1, but not ROS1 Kinase. These findings are significant since elevated levels of HIF-1, iNOS, and mTOR are closely linked to the aging process and damage to the dermal matrix. Consequently, this connection implies a potential strategy to impede cellular aging. Notably, Epicatechin, an active constituent of rCSE, displayed a more effective collective inhibition of iNOS, HIF-1, and rapamycin mTOR compared to the combined effects of the three individual drugs in the control group, as displayed in Table 3 regarding inhibitory or binding values. Furthermore, Kaempferol and Quercitrin, also detected in rCSE, more potently inhibited iNOS and rapamycin mTOR compared to the controls S-ibuprofen and Quercetin. Conversely, in contrast to rCSE, aCSE only featured two compounds with the potential for inhibiting iNOS and rapamycin mTOR: (+)-Catechin and Naringin (Table 3). The ten compounds identified in CS demonstrated lower values in inhibiting ROS1 Kinase, likely due to luteolin’s stronger structure and activity in inhibiting it.

In pharmacoinformatics or molecular docking simulation, CSE has potential as an antioxidant and anti-aging agent, and this is validated in vitro through evidence of its activity in combating free radicals through DPPH and ABTS prevention. See Table S3 in the Supplemental File for a comprehensive representation of amino acid interactions with other substances.

### 2.3. Antioxidant Capabilities of Two Coffee Silverskin Extracts

**Figure 1:**
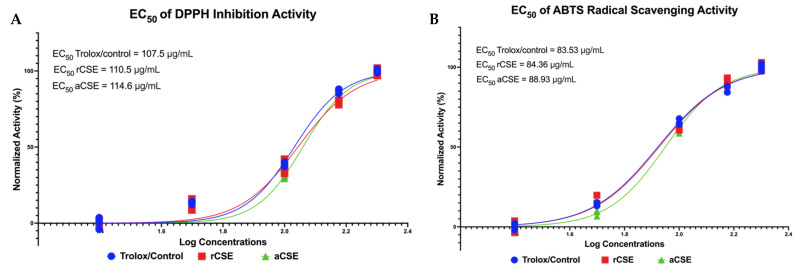
Antioxidants capabilities of coffee silverskin extract (CSE). (**A**) EC_50_ of DPPH inhibition activity by CSE. (**B**) EC_50_ of radical scavenging activity by CSE. rCSE: Robusta-coffee silverskin extract; aCSE: Arabica-coffee silverskin extract.

Determination of DPPH and ABTS inhibition is very important to see the antioxidant potential of CSE, especially to see its potential in inhibiting free radicals in the aging process. Antioxidant activity tests have been carried out on each CS, rCSE, and aCSE using the Trolox control, and the EC_50_ data were summarized in Figure 1. Based on the EC_50_ determination, rCSE has more potent antioxidant activity compared to aCSE (EC_50_ rCSE < aCSE values). However, when further compared to the Trolox control, rCSE has a greater EC_50_ value and this indicates that Trolox is still a leading antioxidant potent based on EC_50_ value. However, a follow-up test was carried out, namely MANOVA on all samples and all concentration gradients.

MANOVA results contained in the Supplementary File (Tables S1 and S2) showed no significant difference (*p* > 0.05) between antioxidant activity control of Trolox, rCSE, and aCSE in both DPPH inhibition and ABTS radical scavenging activity. This shows that the antioxidant ability of the two CSs is still in the range of or equivalent to strong control or Trolox in fighting free radicals and indicates that the antioxidant potential of EC_50_ is slightly different. Furthermore, in DPPH, rCSE has the same activity (not significantly different) as Trolox at concentrations of 25, 50, 100, and 200 μg/mL while aCSE only at 50 μg/mL concentration has the same activity as the control. In ABTS radical scavenging only rCSE appears to be at concentrations of 50, 100, and 200 μg/mL equivalent in activity to Trolox as control. This is based on Dunnett’s multiple comparisons (MANOVA) statistical test conducted between the control group and the CS or samples group, and the data are summarized in Supplementary File Tables S1 and S2. 

### 2.4. In Vitro Modulation of mTOR/AMPK/SIRT1 by Coffee Silverskin Extract

**Figure 2:**
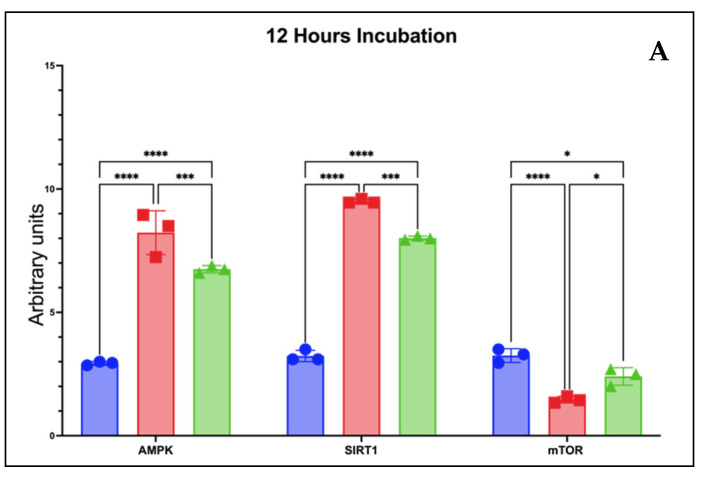

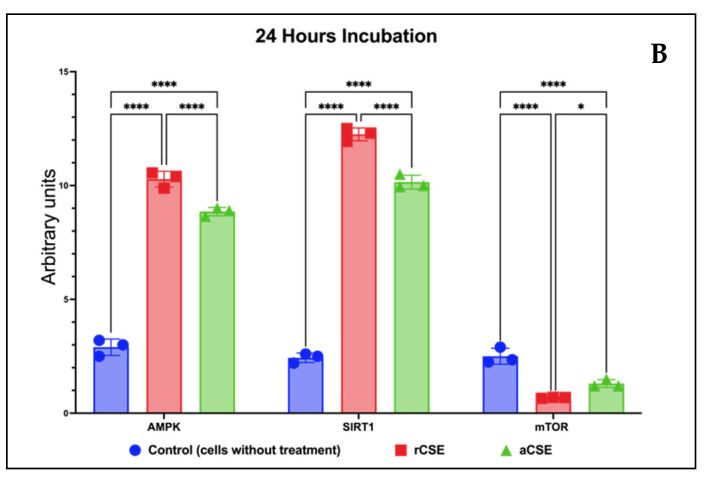
Modulation of AMPK/SIRT1/mTOR expressions by coffee silverskin extract (CSE). (**A**) Expression at 12 h of incubation. (**B**) Expression at 24 h of incubation. **** *p* < 0.0001, *** *p* = 0.0002, * *p* = 0.0105. rCSE: Robusta-coffee silverskin extract; aCSE: Arabica-coffee silverskin extract.

Changes in protein expression of the mTOR/AMPK/SIRT1 pathway were also observed in vitro, and the data from MANOVA analysis are in Figure 2. Figure 2A shows significant activity (*p* < 0.05) of both CS in upregulating of AMPK/SIRT1 and downregulating of mTOR expressions at 12 h incubation. Interestingly, in rCSE which is also aligned with the results of its antioxidant activity, it is more significant in modulating the mTOR/AMPK/SIRT1 pathway at 12 h incubation. Still in line with the modulation results at 12 h of incubation, both CS showed modulation activity of AMPK/SIRT1/mTOR expressions and rCSE still led it at 24 h of incubation (Figure 2B). 

## 3. Discussion

CS, which is the sole byproduct generated during the coffee roasting process, has a global production volume of approximately 76 million kilograms per year [18]. Even though CS is a waste, CS has considerable nutritional properties as it was claimed to become a source of proteins (16%), potassium, magnesium, calcium, and vitamin C that is low in fat (0.44%) and high in fiber (22%) [19]. Moreover, CSEs have been utilized in various aspects of health due to their chemoprotective [12], longevity elevation [11], and metabolic-improving properties [20]; all of them were correlated with antioxidant capabilities. Therefore, it is important to elucidate the antioxidant properties of a functional food source, which are strongly linked with its metabolite profile. Furthermore, a new trend has been observed regarding the modulation of mTOR/AMPK/SIRT1 by functional food in the regulation of the aging process [21]. Taking into account all the mentioned reasonings, this study presents novel insights regarding the metabolite profile, antioxidant activity, and anti-aging potential of CS through in silico and in vitro approaches.

CS is a warehouse of bioactive compounds. Recent research has highlighted that a total of 18 bioactive phenolic compounds and 12 alkaloid compounds were found in CSEs [16]. However, the untargeted metabolomic profiling in this study only revealed four phenolic and seven flavonoid compounds. Next, the molecular docking identifies that Epicatechin, Kaempferol, Quercitrin, (+)-Catechin, and Naringin have greater binding affinity to 3E7G and 3FAP, implying that these compounds may exert effects on iNOS and mTOR receptors. Specifically, Epicatechin reduced the expression of iNOS proteins by downregulating the nuclear factor-kB [22] and also regulating the AMPK and Akt/mTOR signaling pathways [23]. Kaempferol demonstrated the ability to finely regulate the expression of the iNOS gene by effectively curbing the activity of NF-κB. Furthermore, it exhibited a remarkable capacity to thwart the age-linked activation of NF-κB by specifically inhibiting the activity of nicotinamide adenine dinucleotide phosphate oxidase (NADPH oxidase). This process is also induced by advanced glycation end products (AGEs) [24,25]. Aside from that, while Quercitrin and Naringin have been acknowledged to have several biological activities mainly as antioxidant and anti-aging [26,27], no discussions are pointing to the influence of Naringin and Quercitrin on the iNOS and mTOR proteins, declaring that the findings in this study are novel.

In the case of 1H2N, only Epicatechin was shown to exhibit greater binding affinity to the HIF-1α receptor. The beneficial effects of Epicatechin on cardiometabolic risk factors—which are strongly related to HIF-1α—have been reported [28]. However, a previous study also highlighted the possible adverse effect of Epicatechin gallate which activates HIF-1 possibly through the chelation of iron [29]. Therefore, it is interesting to discover that HIF-1α can be a therapeutic target by Epicatechin. Recent research suggests that hypoxia-inducible factor-1α (HIF-1α) has emerged as a crucial transcription factor involved in age-related conditions, specifically in the regulation of cellular senescence associated with cardiovascular aging [30]. HIF-1α is activated when oxygen levels are low which regulates several cellular processes associated with aging, including cellular senescence, oxidative stress response, inflammation, and metabolism [31]. HIF-1α also influences tissue repair and regeneration through the production of growth factors and vascularization.

The findings in this study regarding CSEs’ antioxidant activity highlighted that rCSE has greater antioxidant properties compared to aCSE, as characterized by the lower EC_50_ value. This finding reinforces the previous statement that Robusta coffee has a higher antioxidant capacity and total phenolic content than Arabica [32]. However, the antioxidant capacities of the two CSEs were lower than Trolox (control). The difference in antioxidant activity of both CSEs reported in this study may be attributed to the difference in the solvent used in this study. For instance, the alkaloid extract of *Sonchus oleraceus* yields the highest scavenging activity in ABTS assay while the methanolic extract has the highest inhibitory effect against acetylcholinesterase [33]. Aside from that, the geographical origins of CSEs may result in different metabolite profiles and functional parameters [34].

Empirical evidence distinctly establishes the significant roles assumed by mTOR, AMPK, and SIRT1 within the intricate framework of aging. The mammalian target of rapamycin (mTOR) commands a central position, intricately coordinating cellular metabolic machinations by harmonizing nutrient sensing with a diverse array of core cellular processes. These encompass proteostasis maintenance, autophagy regulation, precise mitochondrial function oversight, modulation of cellular senescence dynamics, and regulation of the progressive waning of stem cell regenerative capacities [35]. Together, these functions contribute to cell growth and proliferation. Furthermore, the hypoactivation of mTOR has been linked with the prevention of aging [36]. This fact corresponds with the finding of this study that rCSE and aCSE significantly reduce the expression of mTOR (*p* < 0.05).

AMPK and SIRT1 are two essential regulatory proteins that play major functions in the aging process. AMPK is activated in response to insufficient energy levels and is involved in cellular energy metabolism. It regulates processes such as glucose and lipid metabolism, mitochondrial function, and autophagy to help maintain cellular homeostasis [37]. AMPK activation has been linked to extending lifespan and delaying age-related diseases. SIRT1 is a member of the Sirtuin protein family and functions as a NAD+-dependent deacetylase. SIRT1 is implicated in numerous cellular processes, such as DNA repair, inflammation, apoptosis, and stress response [38]. It also plays a crucial role in regulating energy metabolism and has been linked to increased lifespan and improved healthspan. Therefore, it can be concluded that AMPK and SIRT1 expressions result in the improvement of health, as the CSEs were shown to significantly elevate the expressions of AMPK and SIRT1 in this study (*p* < 0.05).

The AMPK/SIRT1/mTOR pathways are intricately connected and play crucial roles in regulating cellular metabolism, energy balance, and the aging process. These pathways are considered key players in modulating lifespan and promoting healthy aging. The link between these pathways and aging lies in their interplay and cross-regulation. AMPK and SIRT1 can modulate mTOR activity, thereby influencing cellular processes associated with aging. AMPK activation inhibits mTOR signaling, leading to a decrease in protein synthesis and an increase in autophagy [39]. This process promotes cellular recycling and clearance of damaged components, which is beneficial for cellular health and longevity. SIRT1 can also inhibit mTOR signaling indirectly. It deacetylates and activates the tumor suppressor protein p53, which in turn inhibits mTOR activity [40]. Additionally, SIRT1 promotes autophagy through the deacetylation and activation of autophagy-related proteins. Collectively, the activation of AMPK and SIRT1 and the inhibition of mTOR lead to a state of energy conservation, enhanced cellular maintenance processes like autophagy, and improved stress resistance, all of which are associated with increased lifespan and improved healthspan [41,42]. These pathways interact with each other and with other signaling networks to orchestrate complex cellular responses to energy status and environmental cues, ultimately influencing the aging process (see Graphical Abstract). Therefore, targeting these pathways may become the major upcoming trend in the development of functional food [4].

This study underscores the potential of rCSE and aCSE as promising functional food candidates endowed with anti-aging properties. In addition to the in vitro and silico experimentation undertaken within the current study, it is imperative to undertake further in vivo assessments and human clinical trials to substantiate the health-enhancing effects attributed to CSEs. For future investigations, the incorporation of Western blotting techniques in in vitro analyses could offer supplementary molecular insights to complement the existing results. In preparation for animal trials, forthcoming research must incorporate a meticulous purification process to ensure a purity level surpassing 95% for each discerned metabolite, a validation that can be verified through meticulous NMR and FTIR analyses. The existing limitation in our study lies in the lack of this purification step. To surmount this constraint, the authors intend to integrate the purification protocol and subsequent animal testing into forthcoming animal trials or preclinical studies. This comprehensive approach aims to enhance the credibility and robustness of our findings.

## 4. Material and Methods

### 4.1. Chemical and Instrument

Solvents like ethanol (EtOH), formic acid, and acetonitrile were employed in the preparation and analysis of coffee silverskin extract (CSE) via ultrasound-assisted extraction (UAE) and HPLC-HRMS/ESI-MS. Potassium persulfate (K_2_S_2_O_8_) was essential for the preparation of the ABTS radical scavenging activity assay, while Trolox served as a positive control in antioxidant activity assays. Sodium Dodecyl Sulfate (SDS) Sample Buffer containing tris-HCl, glycerol, β-mercaptoethanol, and bromophenol blue was used for sample preparation in SDS-PAGE. The study also utilized β-Mercaptoethanol and skimmed dry milk in various steps. Antibodies, including Anti-SIRT1, Anti-phospho-mTOR, Anti-phospho-AMPK, and Total-mTOR antibodies, were crucial for protein analysis. Additionally, Goat Anti-Rabbit Secondary Antibodies were employed for detecting specific proteins. Chemical reagents such as DPPH and ABTS were used in antioxidant activity assays.

This study relied on a range of specialized instruments and equipment. The ultrasonic bath or sonicator (Branson 2510 model) was used for ultrasound-assisted extraction (UAE) of CSE. The Thermo Scientific Dionex Ultimate 3000 RSLC Nano HPLC apparatus, equipped with a Hypersil GOLD aQ 50 column, facilitated the HPLC-HRMS/ESI-MS analysis of compounds in CSE. The Thermo Scientific Q Exact apparatus was employed for high-resolution mass spectrometry (HRMS). A spectrophotometer was used for measuring optical density (OD) in protein expression analysis. The ChemDraw Ultra software was utilized for creating and visualizing ligand 2D structures. Computational infrastructure was provided by an ASUS Vivobook M413ia–Ek502t laptop with specific specifications. The study also utilized software tools such as AutoDock Tools (version 4.2) and BIOVIA Discovery for molecular docking simulations and analysis. Access to protein structures was granted through the Protein Data Bank (PDB) and molecular structure information via PubChem. The IkawaTM coffee roaster was essential for roasting coffee beans, while nitrogen was used in the pulverization of silverskin and storage of samples.

### 4.2. Preparation of CS Extract (CSE) via Ultrasound-Assisted Extraction (UAE) Method

**Figure 3:**
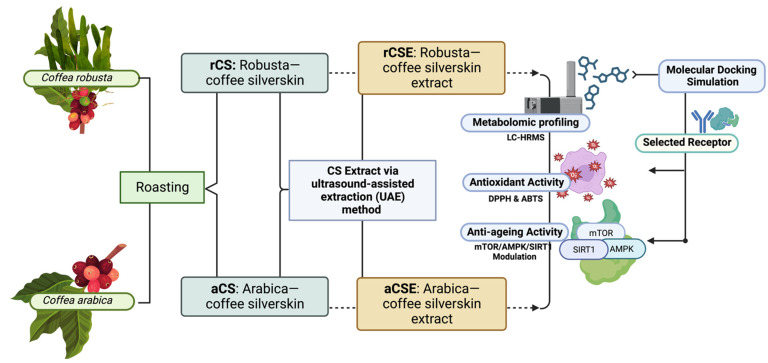
Methodical schematic of CS study flow. Created with BioRender.com Premium License by Fahrul Nurkolis.

This extraction method alludes to well-established publications with minimal modifications [16]. CS was obtained by roasting green beans of *Coffea arabica* and *Coffea robusta* that had been authenticated as originating from Kepuharjo, Cangkringan District, Sleman Regency, Yogyakarta Special Region 55583 (−7.6007304° S latitude, 110.4483609° E longitude), Indonesia. In a previous study, ultrasound-assisted extraction (UAE) was determined to be the optimal technique for identifying compounds in CSE. The coffee was roasted for nine minutes at a maximum temperature of 195 °C using an IkawaTM coffee roaster manufactured by IKAWA Ltd. The silverskin was then pulverized into a fine powder under nitrogen and stored at 4 °C until extraction. Next, 20 g of simplica CS powder was sonicated with 100 mL ethanol using a sonicator (400 W, Branson 2510 model; Danbury, CT, USA) at a frequency of 40.00 kHz for 2 h at 20 °C. Each sample was filtered with filter paper, and the extract obtained was lyophilized. Then, 10 mg of the lyophilized extract was dissolved in 10 mL of 70% ethanol (EtOH: water = 70: 30; 1 mg/mL) and sonicated for 10 min. The CSE results (rCSE: Robusta-coffee silverskin extract; aCSE: Arabica-coffee silverskin extract) were then stored in aluminum foil at −20 °C in dark conditions until further metabolomic profiling and in vitro experiments (Figure 3).

### 4.3. Analyzing Untargeted Metabolomic Data Using HPLC-HRMS/ESI-MS 

The untargeted metabolomic profiling test was performed to analyze the compounds present in the CSE (aCSE and rCSE), which has received laboratory service accreditations for ISO 9001:2008 and ISO 17025 [43]. A volume of 50 µL from each sample was mixed with 96% ethanol and underwent 30 cycles of vortexing. Subsequently, a centrifugation process lasting 2 min was conducted at a speed of 6000 revolutions per minute (rpm). Before conducting the study, the supernatants underwent filtration using a 0.22 µm syringe filter. 

The LC-HRMS system employed in this study comprised a Thermo Scientific Dionex Ultimate 3000 RSLC Nano HPLC apparatus equipped with a micro flow meter. The separation operation was performed utilizing a Hypersil GOLD aQ 50 column, which had dimensions of 50 mm in length and 1 mm in diameter, and a particle size of 1.9 µm that was kept at 30 °C. Solvent A, composed of 0.1% formic acid in water, and solvent B, composed of acetonitrile, were employed in the process. The separation of compounds was achieved by employing a linear gradient with a flow rate of 40 µL/min for a period of 30 min. The Q Exact apparatus manufactured by Thermo Scientific was employed to conduct high-resolution mass spectrometry (HRMS). The instrument exhibited a comprehensive scanning resolution of 70,000 for both positive and negative ionization modes. Additionally, it possessed a data-dependent MS2 resolution of 17,500. The investigation conducted on the studied substances by the HRMS provided a comprehensive and precise dataset.

### 4.4. Pharmacoinformatic Approach via Molecular Docking Simulations

The research was carried out using an ASUS Vivobook M413ia–Ek502t laptop, which boasted specifications such as a 2.3 GHz AMD Ryzen 5 4500u processor, 8 GB DDR4 memory, and a 512 GB M.2 SSD storage. Operating on the Windows 10 Home OS, this laptop provided the computational infrastructure essential for the conducted simulations.

Integral to the research were specific software tools. The docking simulations and ensuing analyses were facilitated by AutoDock tools version 4.2. To create and visualize ligand 2D structures, ChemDraw Ultra version 12.0 was employed. Furthermore, the study harnessed the capabilities of BIOVIA Discovery 21.1, a comprehensive software suite renowned for its molecular modeling and simulation prowess. Acquiring essential data involves tapping into external resources. The Protein Data Bank website (https://www.rcsb.org) played a pivotal role in granting access to an extensive array of protein structures. Similarly, the PubChem structure database (https://pubchem.ncbi.nlm.nih.gov) proved invaluable, furnishing a wealth of molecular structure information for the compounds under investigation.

Molecular pairing simulations were carried out in accordance with well-established research standards and prior research practices [44]. The study utilized ligands sourced from two distinct CSE metabolite profiles. Initially, 2D molecular structures were generated through the employment of ChemDraw Ultra 12.0 software. Subsequently, these 2D structures underwent a conversion into 3D configurations using the MM2 algorithm, thereby enhancing their representation. For target protein acquisition, the Protein Data Bank was accessed. The specific proteins targeted were human inducible mammalian target of rapamycin (mTOR), reactive oxygen species 1 kinase (ROS1), nitric oxide synthase (iNOS), and hypoxia-inducible factor-1 alpha (HIF-1). It is important to note that receptor molecules were assigned Kollman charges, while charges based on the Gasteiger method were allocated to the ligands, a step crucial for subsequent computational analyses.

To ensure the accuracy of the docking procedure, a process called redocking was conducted. This involved the repositioning of the original ligands within the binding pockets of the target proteins, facilitated by the utilization of AutoDock tools (version 4.2). An assessment parameter known as the root mean square deviation (RMSD) was employed to gauge the variance between the original ligand positions and their positions after redocking. A threshold RMSD value of less than 2.0 indicated the reliability of the ligand’s position post-redocking. The determination of docking parameters and grid configurations crucially depended on the outcomes of prior validation procedures. The validation of molecular docking was conducted through a process called redocking. Employing AutoDock tools (version 4.2), the original ligand was relocated to the target binding site using precise grid coordinates. Following the redocking procedure, the RMSD (root mean square deviation) of the ligand’s position needed to be under 2.0 Å for validation. The culmination of the docking simulations resulted in the generation of files with a *dlg extension. These files encapsulated the outcomes of the docking experiments and were subsequently subjected to analysis.

The software employed for the analysis of ligand–receptor interactions was Discovery Studio 2016. Through this platform, the intricate interplay between the ligands and the target receptors could be comprehensively understood and evaluated. This analysis likely provided insights into the binding affinities, orientations, and potential functional implications of the ligand–receptor interactions, contributing to a deeper understanding of the molecular interactions under investigation.

### 4.5. Antioxidants Activities Assay via DPPH and ABTS Inhibition

DPPH radical scavenging activity and ABTS radical scavenging activity were used to test the antioxidant capacities of aCSE and rCSE (Permatasari, Nurkolis, Gunawan, and others, 2022). The DPPH radical scavenging activity test was utilized to determine the level of antioxidant activity. A 3 mL vial that already contained the DPPH reagent was given various doses of Trolox (which served as a control), aCSE, and rCSE. These values were 25, 50, 100, 150, and 200 µg/mL. After letting the mixtures sit at room temperature for half an hour, the absorbance of the samples was measured at 517 nanometers to obtain an accurate reading of the DPPH content.

The last step was to carry out an analysis of the ABTS+ scavenging activity. After preparing a combination of potassium persulfate (K_2_S_2_O_8_; 2.4 mM) and ABTS (7 mM), which was then covered with aluminum foil to shield it from light, the mixture was allowed to react at a temperature of 22 °C for 14 h. The resultant stock solution was diluted (for example, 1 mL of stock solution was added to 60 mL of ethanol [C_2_H_6_O]), which led to the production of a working solution that had an absorbance of 0.706 when measured at 734 nm. At each stage of the evaluation process, novel approaches to problem-solving were developed. Trolox, aCSE, and rCSE samples were combined with the ABTS working solution (1 mL) at various concentrations (25 µg/mL, 50µg/mL, 100 µg/mL, 150 µg/mL, and 200 µg/mL), and absorbance at 734 nm was measured after 7 min.

The proportion of inhibition caused by DPPH and ABTS was determined with the use of a specific formula and written down as a decimal. Each sample was analyzed a total of three times (*n* = 3), which was done so that the results would be as accurate as possible. The antioxidant compound Trolox (C_14_H_18_O_4_) was used as a positive control in both the ABTS and the DPPH tests. The radical scavenging ability of aCSE, rCSE, and Trolox was measured using the half-maximal effective concentration (EC_50_), which was found by our researchers. The sample concentration that results in a decrease of 50% in the initial radical concentration is denoted by the value known as the EC_50_. Utilizing the procedure described in the previous research [45]:

(1) $$\mathrm{Inhibition}\;\mathrm{Activity}\left(\%\right)=\frac{A0-A1}{A0}\times 100\%$$

where *A*0 = absorbance value of blank; and *A*1 = absorbance value of standard or sample.

### 4.6. In Vitro Assay of mTOR/AMPK/SIRT1 Expressions

To generate a mixed mixture of cell lysates with a total concentration of 25 µg/L, the necessary volume of SDS sample buffer was added. Components such as 0.5 M Tris-HCl (pH 6.8), 20% SDS, 10% glycerol (C_3_H_8_O_3_), 5%-mercaptoethanol (HOCH_2_CH_2_SH), and 0.2% bromophenol blue were used in this buffer. After that, this combination was heated for a total of 5 min at a temperature of 95 °C. After this stage, the samples were separated utilizing SDS-PAGE (Sodium Dodecyl Sulfate Polyacrylamide Gel Electrophoresis), and then they were transferred to a polyvinylidene difluoride membrane for further analysis. To detect SIRT1, total AMPK, and mTOR, a polyvinylidene difluoride membrane was treated with a blocking solution that consisted of 5% skimmed dry milk in a buffer that was composed of saline buffered Tris with Tween (T-TBS). This was done to prevent the membrane from absorbing any of the detecting reagents. This buffer had a concentration of 0.1% Tween 20 and contained 20 mmol/L Tris-HCl, 0.138 mol/L Sodium chloride (NaCl), and had a pH of 7.4. On the other hand, to identify phosphorylated forms of AMPK and mTOR, a blocking solution that consisted of 5% albumin (particularly bovine serum albumin or BSA) in T-TBS was used to treat the membrane. This was done so that the phosphorylated forms of AMPK and mTOR could be detected.

To assess SIRT1 expression and mTOR/AMPK activation, a specific methodology was followed. The process included exposing the cellular membrane to primary antibodies, followed by secondary antibodies linked to peroxidase. The primary and secondary antibodies were suitably diluted in a solution containing 5% Bovine Serum Albumin (BSA) within a T-TBS solution. Primary antibodies were allowed to incubate overnight at a temperature of 4 °C, following these specifications: Anti-SIRT1 antibody, derived from rabbits, was used at a 1:1000 dilution. Anti-phospho-mTOR antibody, also from rabbits, was applied at a dilution of 1:1000. Anti-phospho-AMPK antibody, sourced from rabbits, was utilized at a 1:200 dilution. Total-mTOR antibody obtained from rabbits was used at a 1:1000 dilution. Concerning the secondary antibodies, two varying dilutions were employed. Goat anti-rabbit secondary antibody at a 1:10,000 dilution was employed to detect SIRT1 and both total and phospho-mTOR while goat anti-rabbit secondary antibody at a 1:2000 dilution was used for detecting both total and phospho-AMPK.

By adopting this comprehensive antibody-based technique, the study aimed to gain insights into SIRT1 expression and the activation states of mTOR and AMPK, while ensuring precision through proper antibody dilutions and incubation conditions. To supplement the information, the experimental process involved seeding 5000 cells into each well using 100 L of medium. These cells underwent treatment with two distinct CS extracts: rCSE and aCSE, at a concentration of 25 M. The treatment spanned two different time frames—6 and 24 h of incubation. Subsequently, the obtained data were subjected to analysis to ascertain the percentage values relative to the control group, which consisted of cells not subjected to any treatment. The assessment of these percentage values was facilitated through optical density (OD) measurements, which were carried out utilizing a spectrophotometer. The wavelength employed for these measurements was determined based on the specifications outlined in the manufacturers’ protocols specific to the respective instrument used for analysis. Moreover, the in vitro analysis of mTOR, AMPK, and SIRT1 was executed in accordance with both the manufacturers’ protocols and the study’s established experimental guidelines. This comprehensive approach ensured that the evaluations of these specific molecules were conducted using standardized procedures, enabling accurate and reliable results within the scope of the study [4,21,46].

### 4.7. Data Analysis and Management

The statistical analysis in this study utilized the MacBook version of GraphPad Prism 9 Premium Software, developed by GraphPad Software, Inc. located in San Diego, CA, USA. The dataset was represented in terms of mean values accompanied by their corresponding standard deviations, denoted as mean SD. To determine the EC_50_, a specific type of non-linear regression known as “log(inhibitor) vs. normalized response—variable slope)” was applied using the advanced statistical analysis capabilities of GraphPad Premium. The primary objective of this analysis was to comprehensively evaluate the outcomes derived from three distinct in vitro experiments, all of which were conducted in triplicate. The assessment focused on measuring the degree of antioxidant inhibition concerning DPPH and ABTS. Concurrently, the expression levels of SIRT1, AMPK, and mTOR were subjected to scrutiny through a two-way ANOVA (MANOVA) analysis, and a post hoc analysis of Tukey or Dunnet was performed. To ascertain statistical significance, a confidence interval (CI) of 95% was adopted, with the threshold for significance set at a *p*-value below 0.05. This rigorous statistical approach ensured that results achieving this level of significance were deemed to be statistically meaningful within the established parameters.

## 5. Conclusions

The metabolites profile or chemical constituents of two Coffee silverskin extracts (CSE), accompanied by molecular activity against selected age-related oxidants receptors and their antioxidant potential as determined by molecular docking simulation, were obtained and observed in this study. Some of the compounds discovered in CSE have promising potential as iNOS, mTOR, and HIF-1 inhibitors. These compounds are mainly flavonoids and polyphenols, including Epicatechin, Kaempferol, and Quercitrin, which were detected in rCSE, and (+)-Catechin and Naringin, which were detected in aCSE. Intriguingly, additional in vitro biological activity tests of antioxidant and anti-aging activity from CS extract demonstrated the same promising potential as the outcomes of a molecular docking simulation, particularly rCSE or coffee silverskin extract from Robusta coffee, which is more potent than Arabica coffee. Additionally, protein expression modulation in the mTOR/AMPK/SIRT1 pathway by CS extracts indicated their role in regulating aging-related cellular processes. These findings have implications for potential use in skincare products, dietary supplements, or pharmaceuticals, but further research and clinical studies are essential to validate their benefits fully. The prospective efficacy value of rCSE must be determined through in vivo investigations and clinical trials.

## 6. Patents

The preparation method and formulation of CSE (rCSE and aCSE) as a Novel Anti-Aging Functional Food resulting from the work reported in this study have been registered as a patent in Indonesia with number S00202305125 (Fahrul Nurkolis is the patent holder of the CSE Formulation).

## Figures and Tables

**Table 1:** Observed compounds in two coffee silverskin extracts via HPLC-ESI-HRMS/MS analysis.

Sample	Observed Compounds	Molecular Formula	RT (Min)	Observed MW (*m*/*z*)	PubChem ID or Substance ID	CAS Number	Type
aCSE	Epicatechin	C_15_H_14_O_6_	16.07	289.8855	72276	490-46-0	Flavonoids
	Kaempferol	C_15_H_10_O_6_	10.14	285.9800	5280863	520-18-3	Flavonoids
	Quercitrin	C_21_H_20_O_11_	9.02	448.1100	5280459	522-12-3	Flavonoids
	4-Hydroxycinnamic acid	C_9_H_8_O_3_	19.55	164.5500	637542	501-98-4	Polyphenols (Phenolic Acids)
	Gallic acid	C_7_H_6_O_5_	16.02	170.1400	370	149-91-7	Polyphenols (Phenolic Acids)
rCSE	Shikimic Acid	C_7_H_10_O_5_	10.90	174.0855	8742	138-59-0	Polyphenols (Phenolic Acids)
	(2R,3S,4S,5R,6S)-2-(hydroxymethyl)-6-[7-hydroxy-3-[(2S,3R,4S,5S,6R)-3,4,5-trihydroxy-6-(hydroxymethyl)oxan-2-yl]oxy-2-(3,4,5-trihydroxyphenyl)chromenylium-5-yl]oxyoxane-3,4,5-triol	C_27_H_31_O_17_^+^	12.92	627.1522	10100906	17670-06-3	Flavonoids
	Caffeic acid	C_9_H_8_O_4_	9.10	179.8800	689043	331-39-5	Polyphenols (Phenolic Acids)
	Naringin	C_27_H_32_O_14_	15.13	580.0300	442428	10236-47-2	Flavonoids
	Rutin	C_27_H_30_O_16_	13.56	610.0225	5280805	153-18-4	Flavonoids
	(+)-Catechin	C_15_H_14_O_6_	14.30	290.0100	9064	154-23-4	Flavonoids

aCSE: Arabica-coffee silverskin extract; rCSE: Robusta-coffee silverskin extract; RT: Retention Time (Minutes); MW: Molecular Weight.

**Table 2:** Validation of molecular docking simulation.

No.	Target Proteins or Receptors	PDB ID	Docking Site (x;y;z)	Docking Area (x;y;z)	RMSD (Å)	ΔG (kcal/mol)	Number in Cluster (/100)	Judgment (<2 Å)
1	iNOS	3E7G	55.022, 21.817, 78.677	40 × 40 × 40	1.789	−6.67	98	Valid
2	mTOR	3FAP	−9.233, 26.776, 35.832	46 × 40 × 42	1.422	−21.75	100	Valid
3	ROS1 Kinase	3ZBF	42.521, 19.649, 3.987	40 × 40 × 40	1.216	−7.83	90	Valid
4	HIF-1α	1H2N	19.984, 25.64, 28.282	40 × 40 × 40	1.128	−3.82	100	Valid

PDB ID: Protein Data Bank ID; RMSD: root mean square deviation.

**Table 3:** Molecular docking parameter of observed compounds.

No.	Substance	Number in Cluster (/100)				dG (kcal/mol)				Ki			
		3E7G	3FAP	3ZBF	1H2N	3E7G	3FAP	3ZBF	1H2N	3E7G	3FAP	3ZBF	1H2N
Control													
1	S-ibuprofen	33				−4.73				128.28 uM			
2	Quercetin		29				−5.96				41.26 uM		
3	Luteolin			96				−6.68				6.86 uM	
4	Genistein				49				−6.77				9.14 uM
aCSE													
1	Epicatechin	92	27	68	61	−6.15	−6.07	−6.10	−7.00	15.85 uM	20.92 uM	14.20 uM	5.59 uM
2	Gallic acid	39	67	87	100	−4.03	−3.95	−3.72	−4.97	710.63 uM	617.81 uM	1.11 mM	86.21 uM
3	4-Hydroxycinnamic acid	55	64	98	99	−4.37	−4.46	−4.95	−5.20	433.26 uM	838.56 uM	180.35 uM	133.46 uM
4	Kaempferol	31	41	100	48	−5.86	−6.17	−6.45	−6.25	39.82 uM	17.09 uM	17.88 uM	24.17 uM
5	Quercitrin	30	24	29	36	−5.41	−7.10	−6.57	−6.07	20.98 uM	2.86 uM	5.08 uM	1.63 uM
rCSE													
1	Caffeic acid	26	74	96	75	−4.19	−4.54	−5.16	−5.13	490.27 uM	303.08 uM	114.81 uM	117.47 uM
2	(+)-Catechin	51	29	43	59	−6.07	−6.48	−6.15	−6.28	33.94 uM	16.65 uM	17.30 uM	19.97 uM
3	(2*R*,3*S*,4*S*,5*R*,6*S*)-2-(hydroxymethyl)-6-[7-hydroxy-3-[(2*S*,3*R*,4*S*,5*S*,6*R*)-3,4,5-trihydroxy-6-(hydroxymethyl)oxan-2-yl]oxy-2-(3,4,5-trihydroxyphenyl)chromenylium-5-yl]oxyoxane-3,4,5-triol	37	9	33	28	−3.98	−5.54	−4.41	−3.39	36.27 uM	19.12 uM	7.57 uM	3.26 mM
4	Naringin	24	16	56	61	−5.78	−8.37	−6.47	−6.37	3.58 uM	82.91 nM	2.59 uM	21.26 uM
5	Rutin	12	8	18	18	−3.79	−5.93	−4.98	−3.38	119.48 uM	12.61 uM	5.66 uM	3.35 mM
6	Shikimic Acid	64	35	82	90	−4.05	−4.22	−3.33	−4.99	525.14 uM	398.89 uM	2.12 mM	128.97 uM

rCSE: Robusta-coffee silverskin extract; aCSE: Arabica-coffee silverskin extract.

## Data Availability

The data sets generated and/or analyzed in this study are available in the manuscript or can be requested from the author (C.H. and F.N.) upon reasonable request via B.K. (Corresponding author).

## Supplementary Materials

The following supporting information can be downloaded at: https://www.mdpi.com/article/10.3390/molecules28207037/s1, Table S1: Two-way ANOVA of DPPH Inhibition Activity; Table S2: Two-way ANOVA of ABTS Inhibition Activity; Table S3: The full visualization from molecular docking simulation of amino acid interactions throughout the observed CSE substance.
