# Supplementary material for: Coffee Silverskin Phytocompounds as a Novel Anti-Aging Functional Food: A Pharmacoinformatic Approach Combined with In Vitro Study
Source: Molecules. 2023 Oct 11;28(20):7037. doi: 10.3390/molecules28207037 (PMC10609341; doi:10.3390/molecules28207037)
Supplement: Supplementary file 1 [file molecules-28-07037-s001.zip › molecules-2578064-supplementary.pdf]

Table S1. Two-way ANOVA of DPPH Inhibition Activity

**DPPH**; Dunnett's multiple comparisons test	Mean Diff.	95.00% CI of diff.	Below threshold?	Summary	Adjusted P Value
**Row 1 or 25 µg/mL**					
Trolox/Control vs. rCSE	-0.5600	-2.860 to 1.740	**No**	**ns**	0.7966
Trolox/Control vs. aCSE	2.233	-0.06640 to 4.533	**No**	**ns**	0.0578
**Row 2 or 50 µg/mL**					
Trolox/Control vs. rCSE	-0.3667	-2.666 to 1.933	No	ns	0.9055
Trolox/Control vs. aCSE	2.733	0.4336 to 5.033	Yes	*	0.0184
**Row 3 or 100 µg/mL**					
Trolox/Control vs. rCSE	0.6000	-1.700 to 2.900	No	ns	0.7711
Trolox/Control vs. aCSE	6.467	4.167 to 8.766	Yes	****	<0.0001
**Row 4 or 150 µg/mL**					
Trolox/Control vs. rCSE	3.577	1.277 to 5.876	Yes	**	0.0022
Trolox/Control vs. aCSE	5.133	2.834 to 7.433	Yes	****	<0.0001
**Row 5 or 200 µg/mL**					
Trolox/Control vs. rCSE	0.06667	-2.233 to 2.366	No	ns	0.9967
Trolox/Control vs. aCSE	2.840	0.5403 to 5.140	Yes	*	0.0142

Table S2. Two-way ANOVA of ABTS Inhibition Activity

Dunnett's multiple comparisons test	Mean Diff.	95.00% CI of diff.	Below threshold?	Summary	Adjusted P Value
**Row 1 or 25 µg/mL**					
Trolox/Control vs. rCSE	3.143	0.6042 to 5.683	Yes	*	0.0140
Trolox/Control vs. aCSE	5.973	3.434 to 8.513	Yes	****	<0.0001
**Row 2 or 50 µg/mL**					
Trolox/Control vs. rCSE	1.970	-0.5692 to 4.509	No	ns	0.1439
Trolox/Control vs. aCSE	8.847	6.307 to 11.39	Yes	****	<0.0001
**Row 3 or 100 µg/mL**					
Trolox/Control vs. rCSE	4.877	2.337 to 7.416	Yes	***	0.0002
Trolox/Control vs. aCSE	6.973	4.434 to 9.513	Yes	****	<0.0001
**Row 4 or 150 µg/mL**					
Trolox/Control vs. rCSE	0.1367	-2.403 to 2.676	No	ns	0.9886
Trolox/Control vs. aCSE	3.773	1.234 to 6.313	Yes	**	0.0032
**Row 5 or 200 µg/mL**					
Trolox/Control vs. rCSE	2.403	-0.1358 to 4.943	No	ns	0.0653
Trolox/Control vs. aCSE	4.400	1.861 to 6.939	Yes	***	0.0007

Table S3. The fully visualization from molecular docking simulation of amino acid interactions throughout the observed CSE substance

iNOS 3E7G

No.	Substance	Visualization
Native Ligand		
1	3E7G	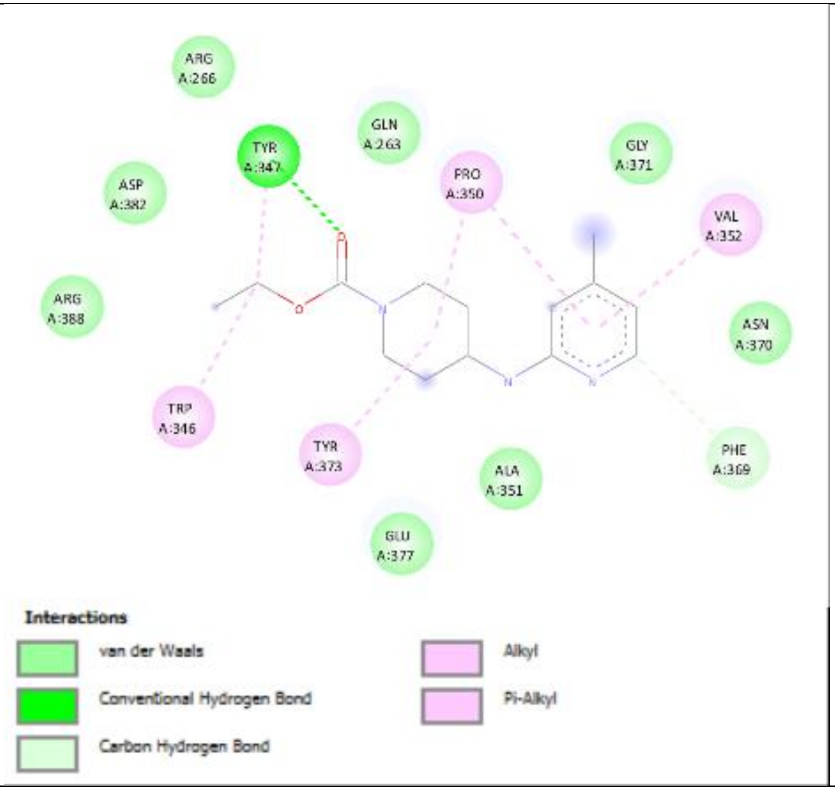
Control		

1	S-ibuprofen	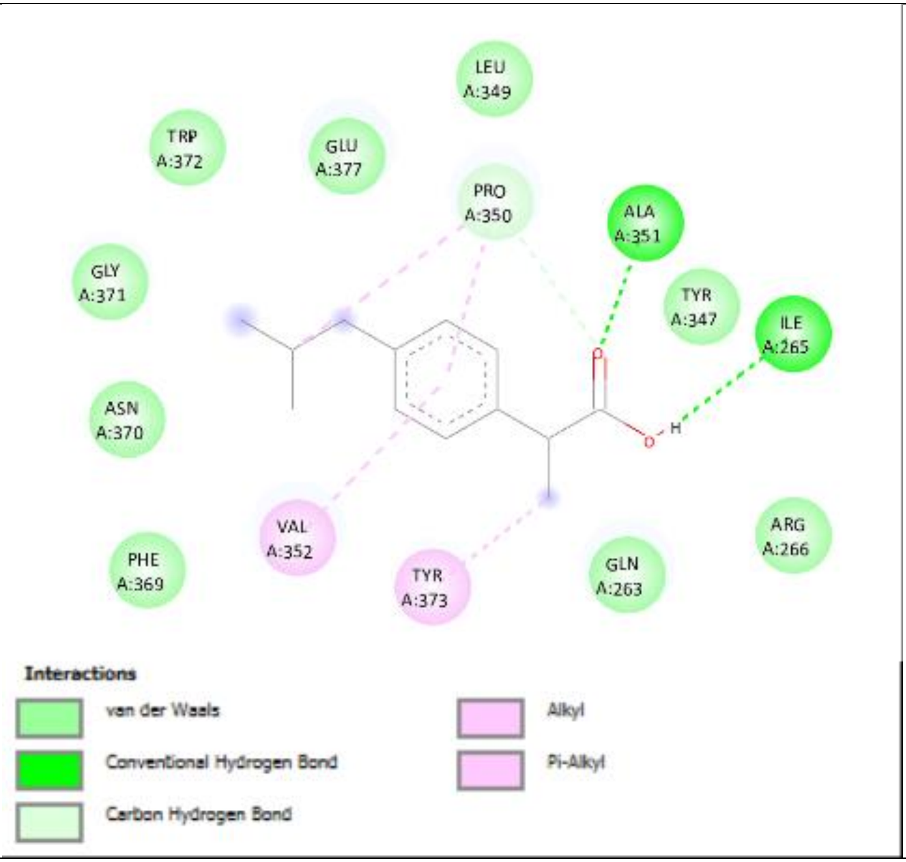
aCSE		

1	Epicatechin	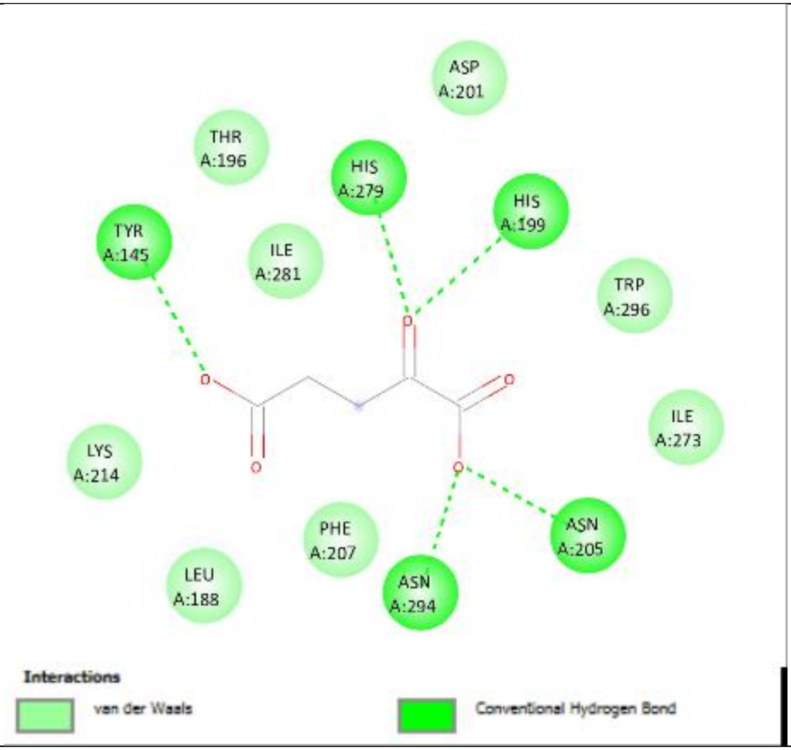	

2	Gallic acid	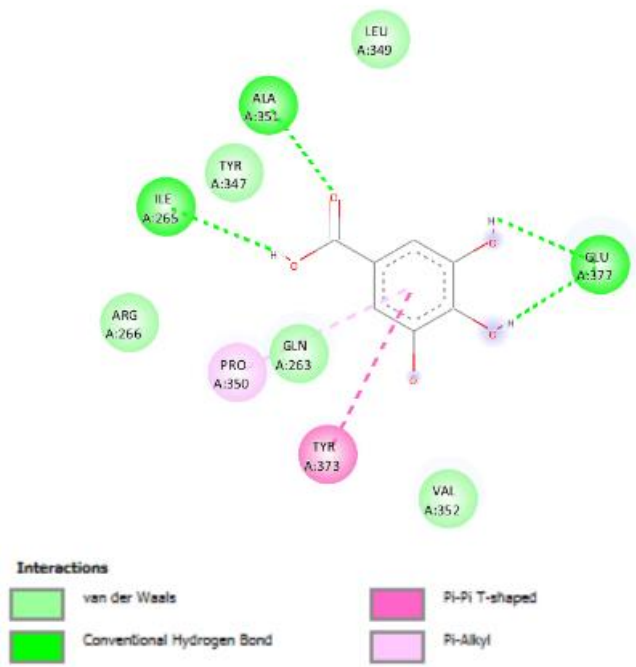

3	4-Hydroxycinnamic acid	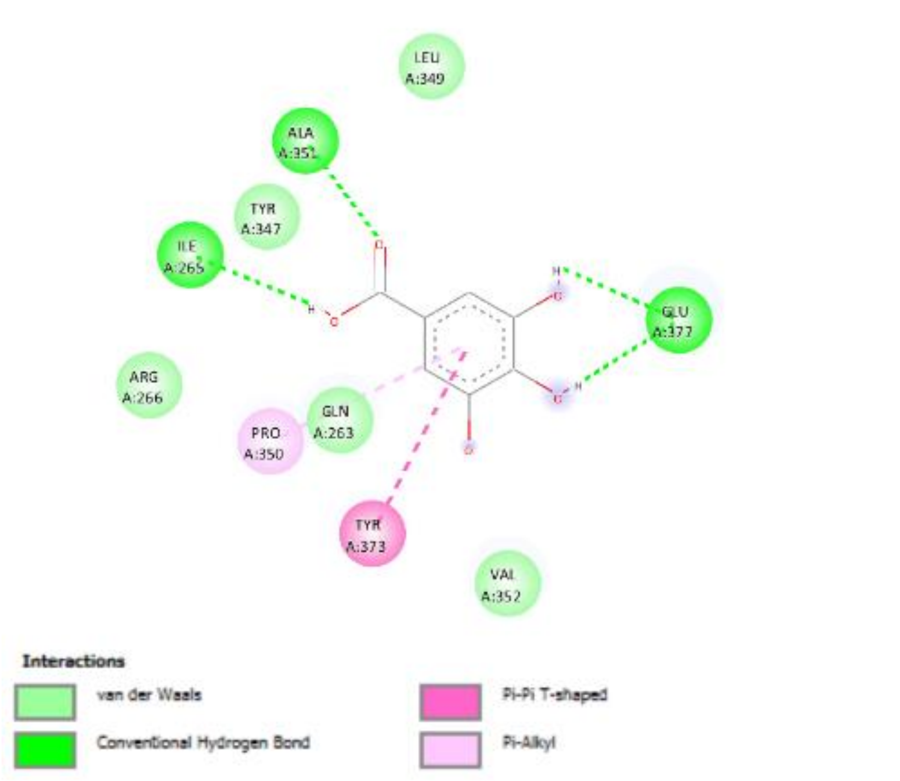

4	Kaempferol	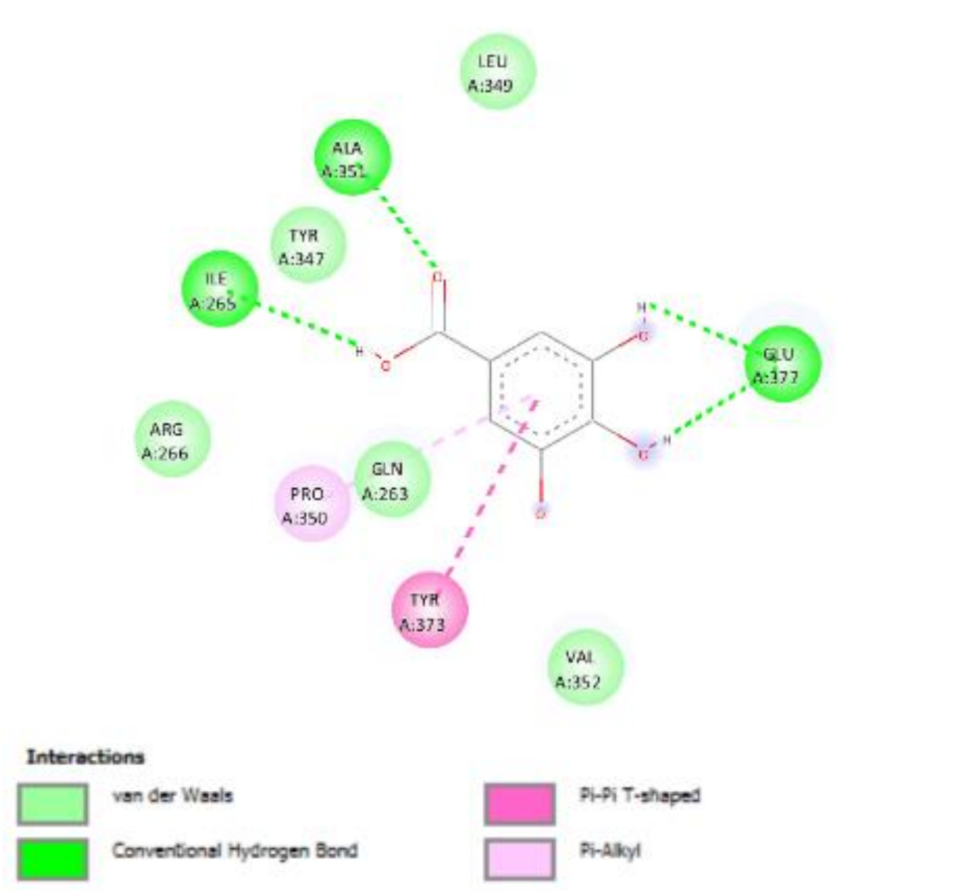

5	Quercitrin	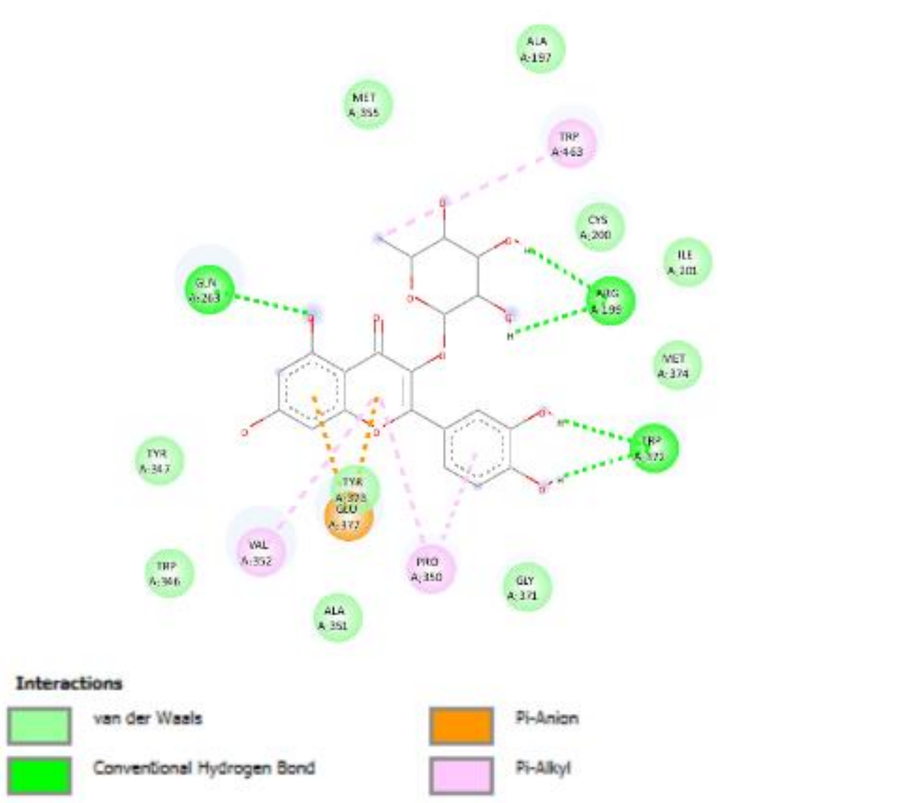
rCSE		

1	Caffeic acid	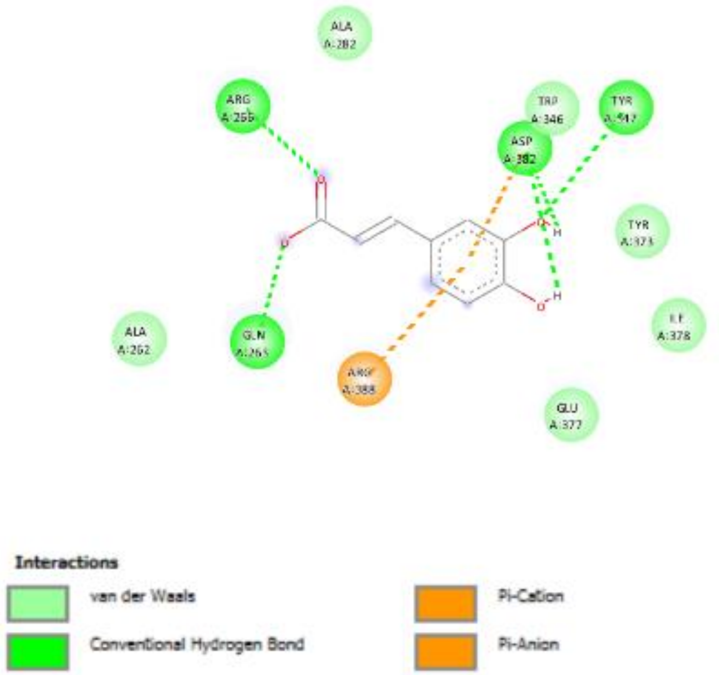

2	(+)-Catechin	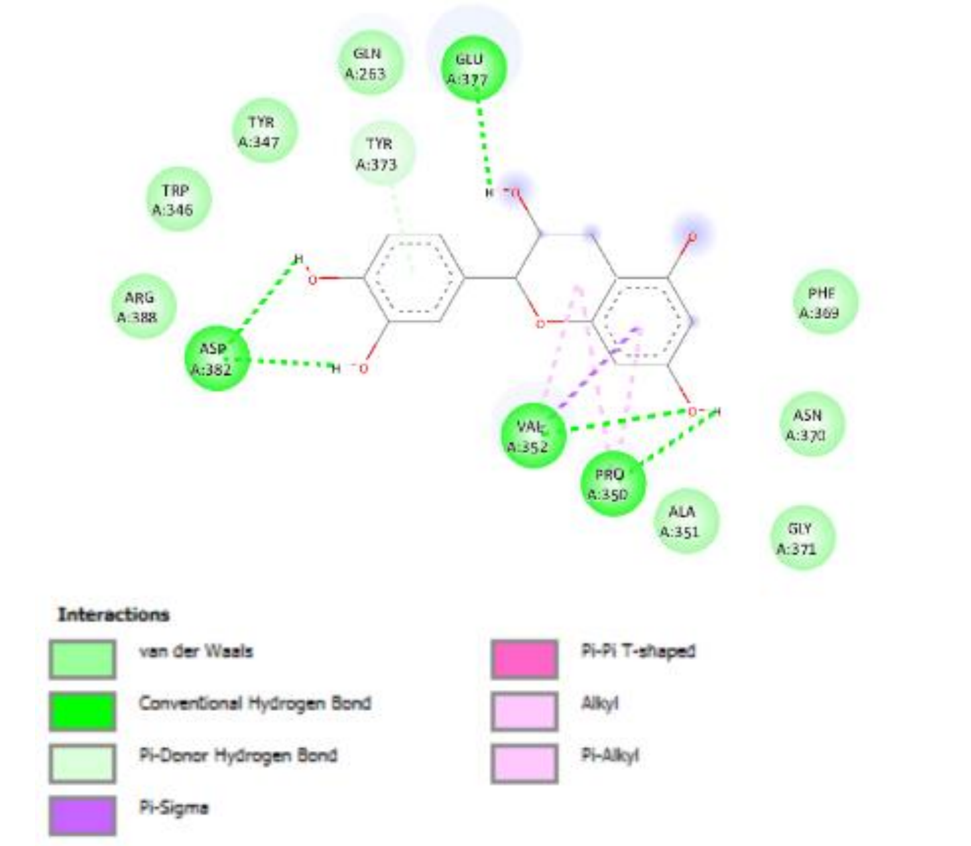GLN A:263<br>GLU A:377<br>TYR A:347<br>TYR A:373<br>TRP A:346<br>ARG A:388<br>ASP A:382<br>PHE A:369<br>VAL A:352<br>ASN A:370<br>PRO A:350<br>ALA A:351<br>GLY A:371<br>Interactions<br>van der Waals<br>Conventional Hydrogen Bond<br>Pi-Donor Hydrogen Bond<br>Pi-Sigma<br>Pi-Pi T-shaped<br>Alkyl<br>Pi-Alkyl

3	(2R,3S,4S,5R,6S)-2-(hydroxymethyl)-6-[7-hydroxy-3-[(2S,3R,4S,5S,6R)-3,4,5-trihydroxy-6-(hydroxymethyl)oxan-2-yl]oxy-2-(3,4,5-trihydroxyphenyl)chromenylium-5-yl]oxyoxane-3,4,5-triol	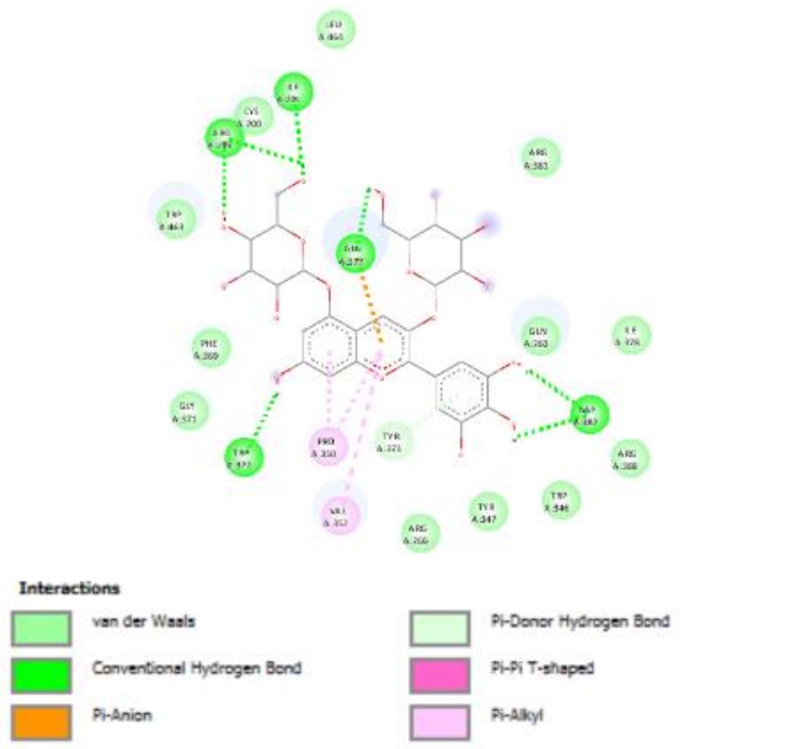

4	Naringin	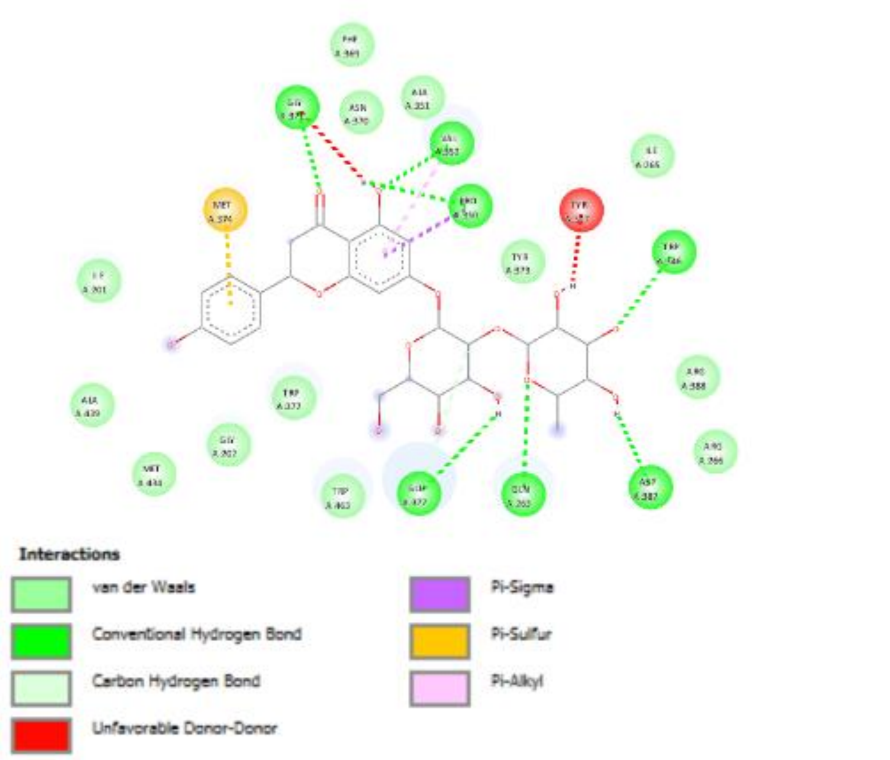 Interactions<br>van der Waals<br>Conventional Hydrogen Bond<br>Carbon Hydrogen Bond<br>Unfavorable Donor-Donor<br>Pi-Sigma<br>Pi-Sulfur<br>Pi-Alkyl

5	Rutin	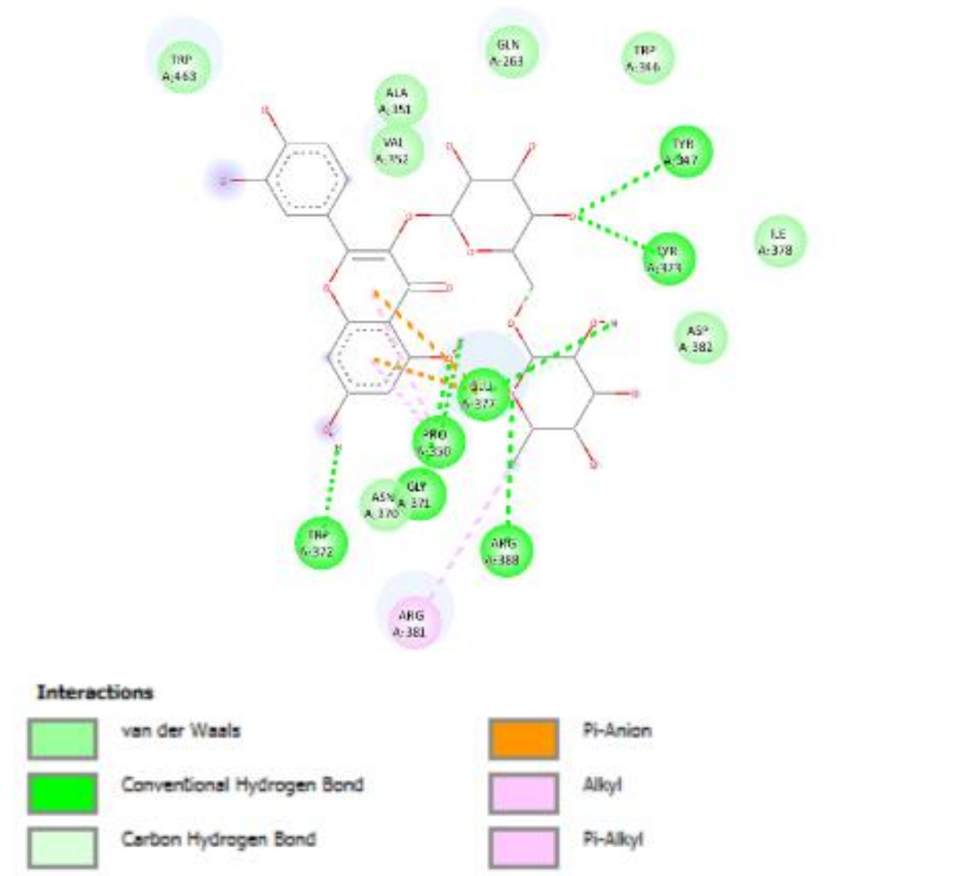

TRP A:463
GLN A:263
TRP A:346
ALA A:351
VAL A:352
TYR A:347
TYR A:473
ILE A:378
ASP A:382
GLU A:377
PRO A:330
ASN A:370
GLY A:371
TRP A:372
ARG A:388
ARG A:381
Interactions
van der Waals
Conventional Hydrogen Bond
Carbon Hydrogen Bond
Pi-Anion
Alkyl
Pi-Alkyl

6	Shikimic Acid	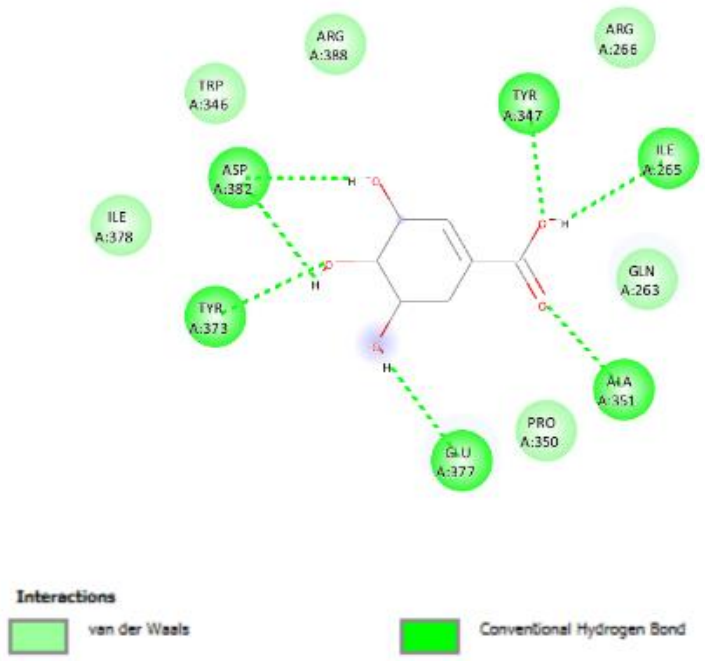

mTOR

No.	Substance	Visualization
Native Ligand		

1	3FAP	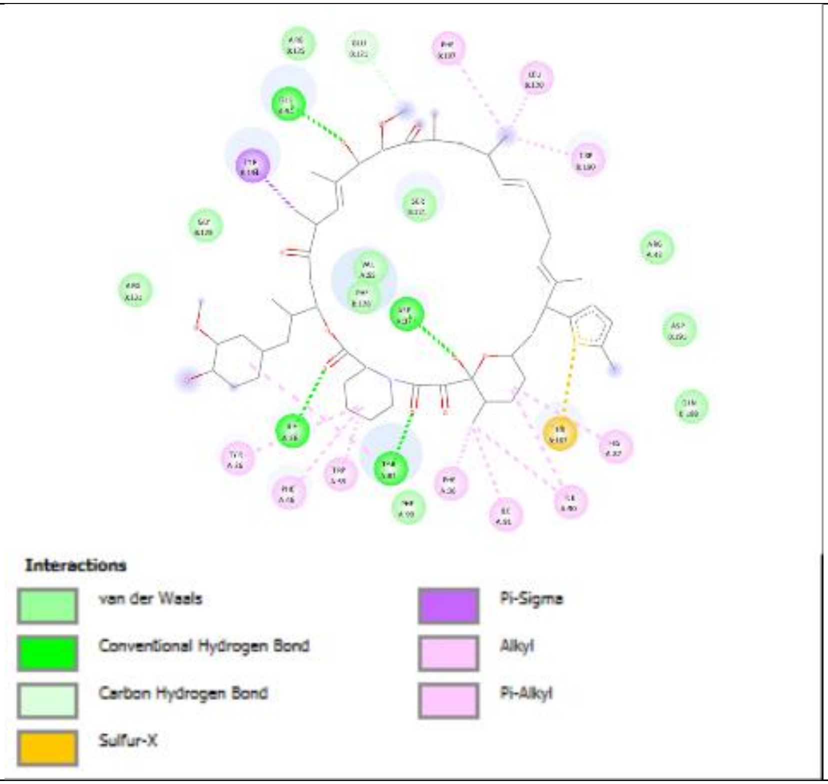	
Control			

1	Quercetin	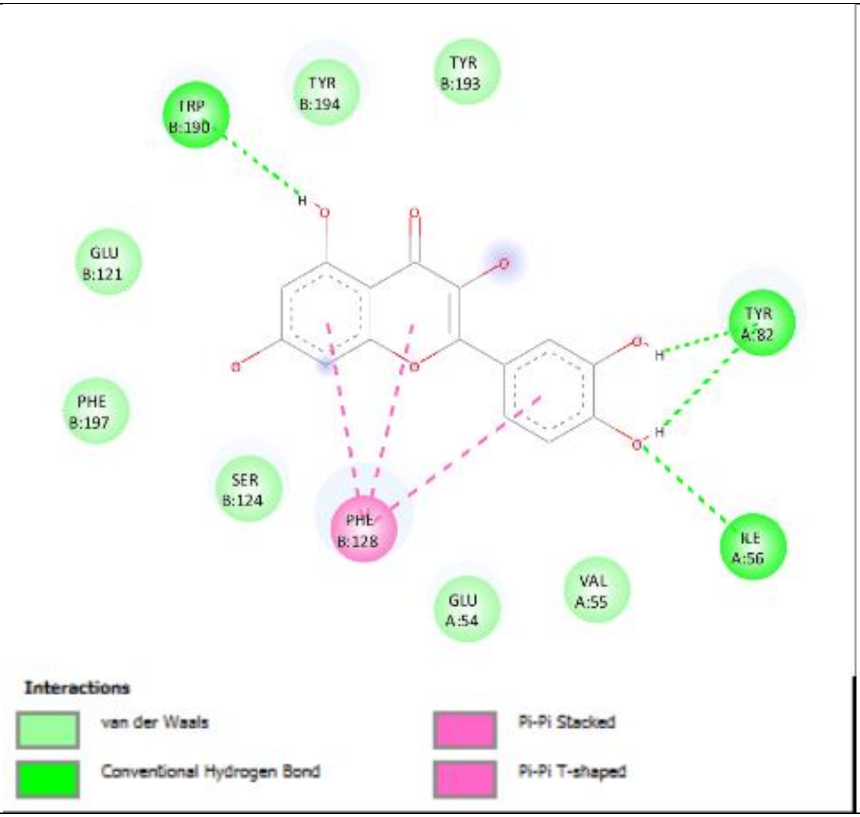	
aCSE			

1	Epicatechin	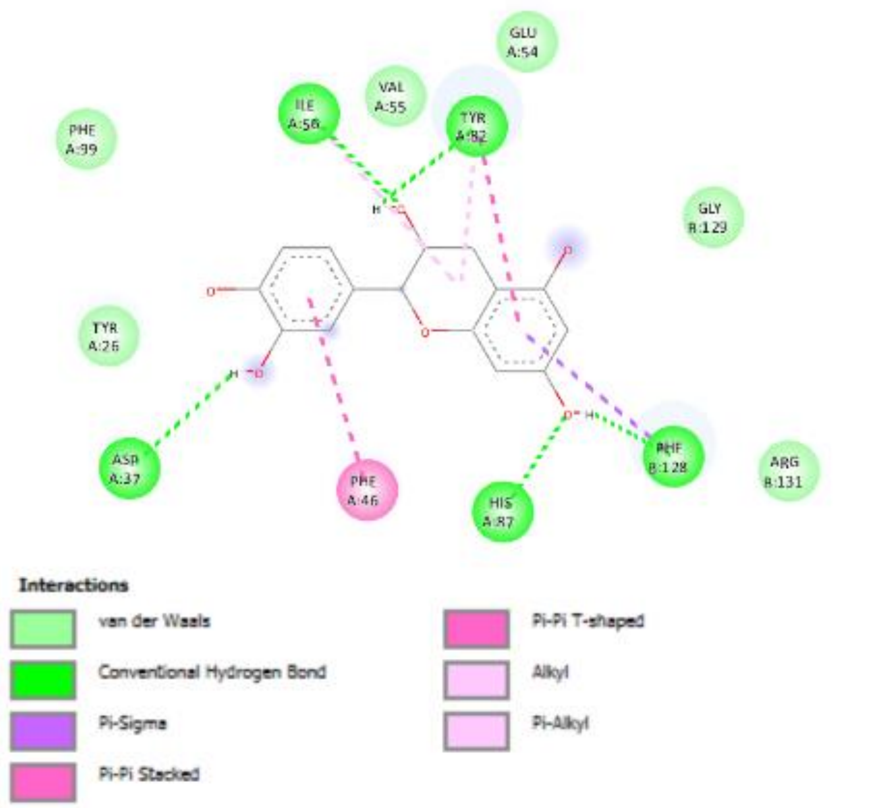

2	Gallic acid	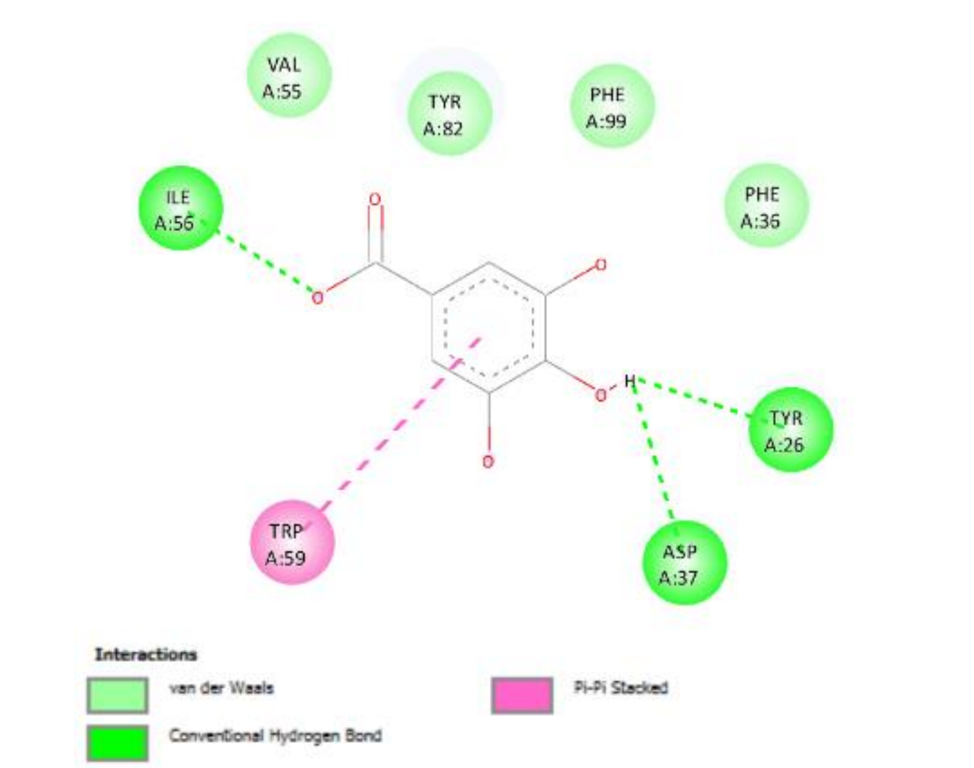

3	4-Hydroxycinnamic acid	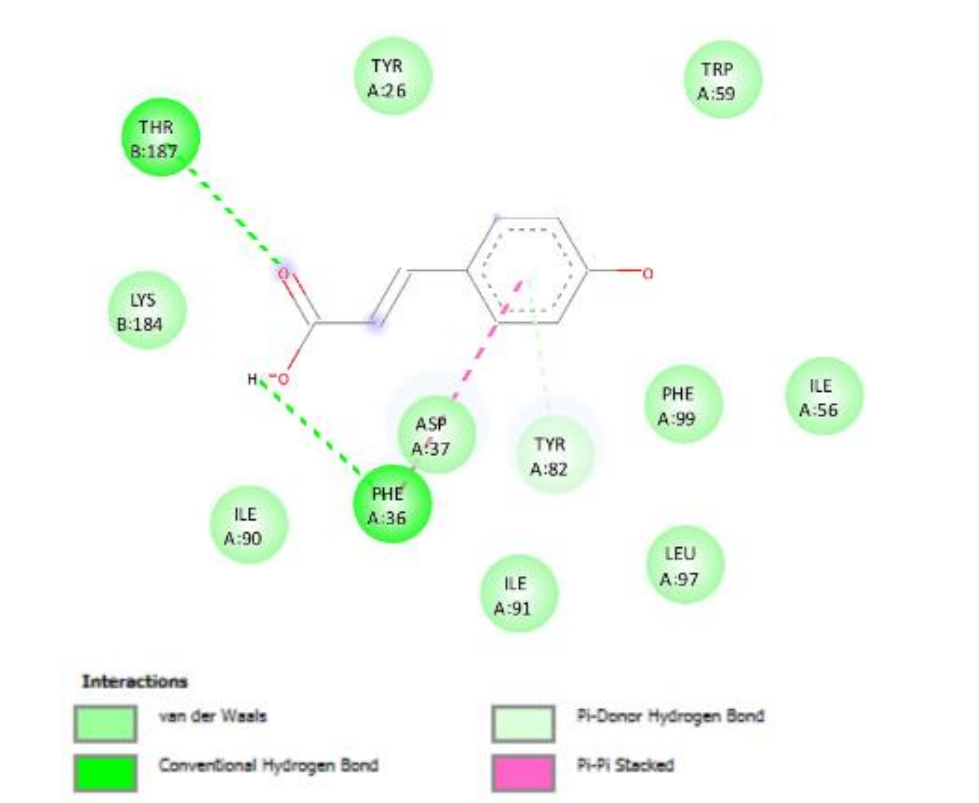

4	Kaempferol	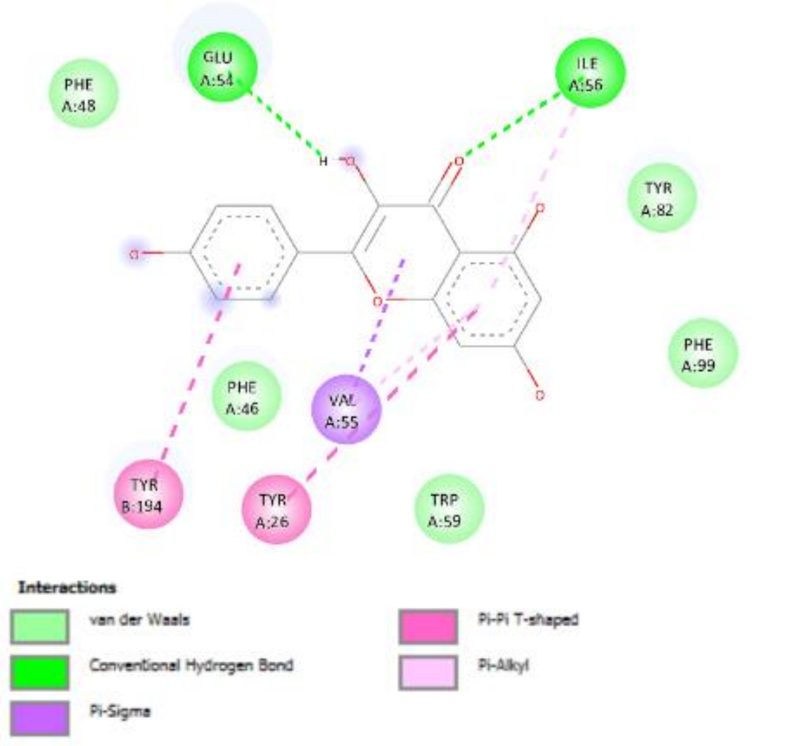

5	Quercitrin	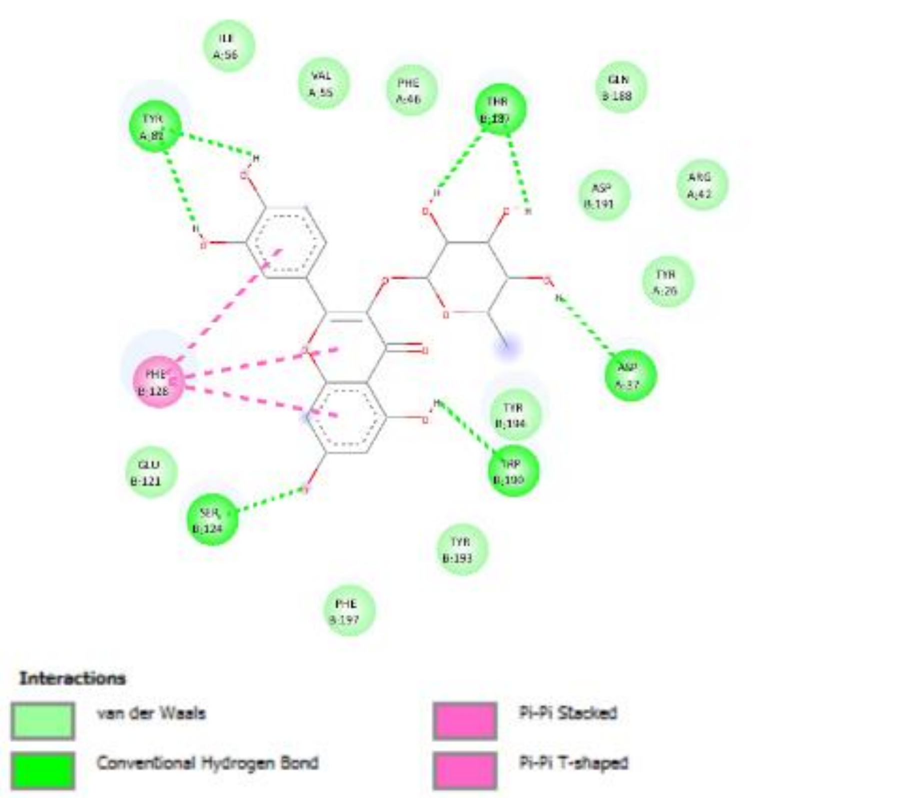
rCSE		

1	Caffeic acid	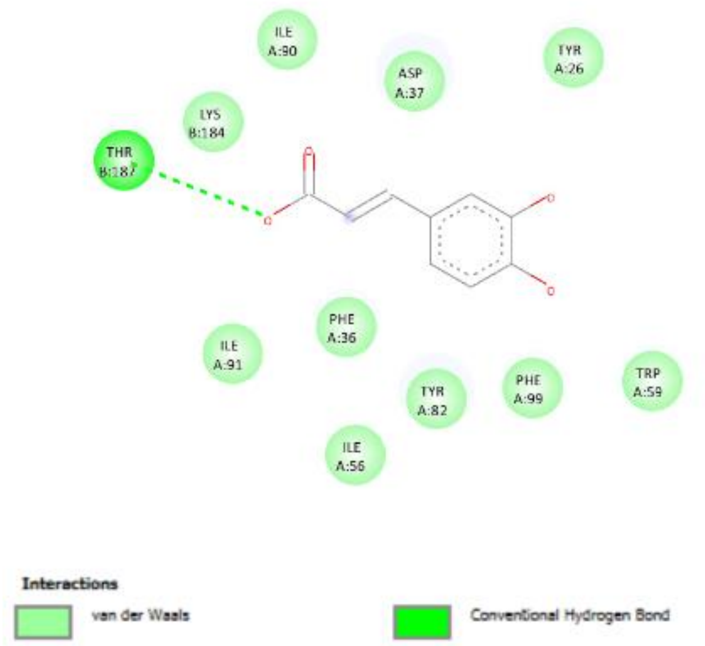

2	(+)-Catechin	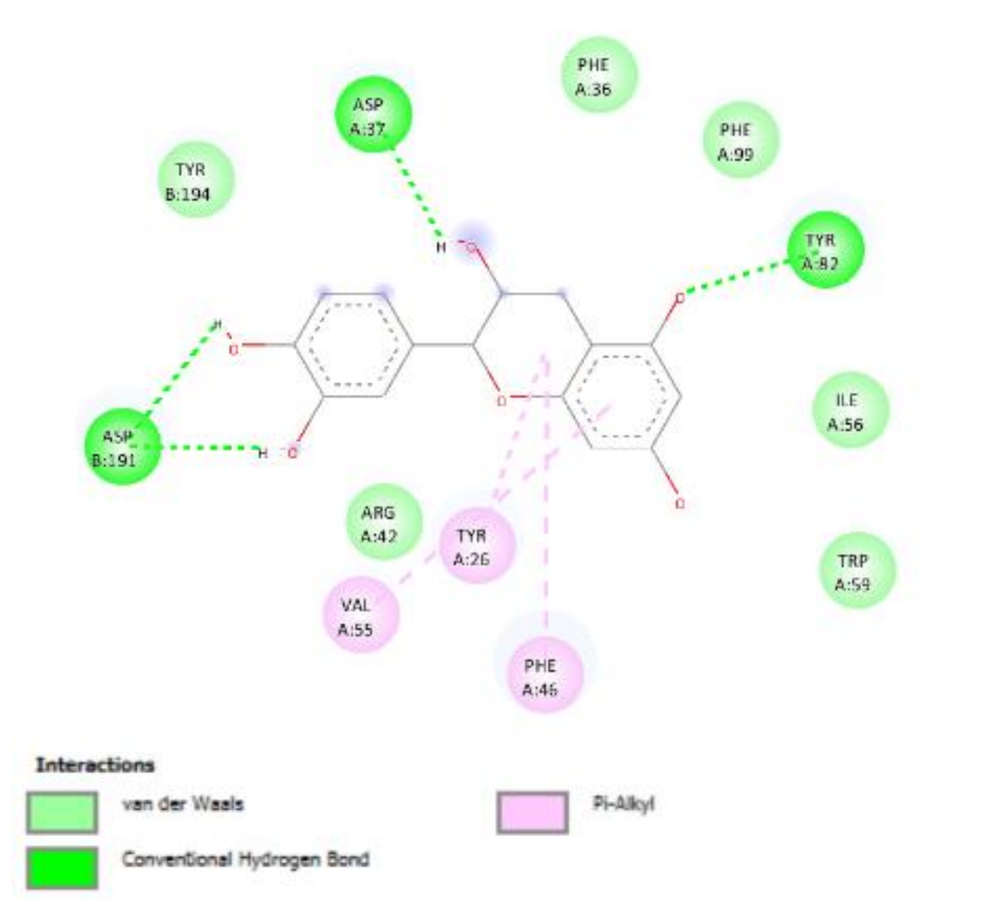

PHE A:36
ASP A:37
PHE A:99
TYR B:194
TYR A:82
ILE A:56
ASP B:191
ARG A:42
TYR A:26
TRP A:59
VAL A:55
PHE A:46
Interactions
van der Waals
Pi-Alkyl
Conventional Hydrogen Bond

3	(2R,3S,4S,5R,6S)-2-(hydroxymethyl)-6-[7-hydroxy-3-[(2S,3R,4S,5S,6R)-3,4,5-trihydroxy-6-(hydroxymethyl)oxan-2-yl]oxy-2-(3,4,5-trihydroxyphenyl)chromenylium-5-yl]oxyoxane-3,4,5-triol	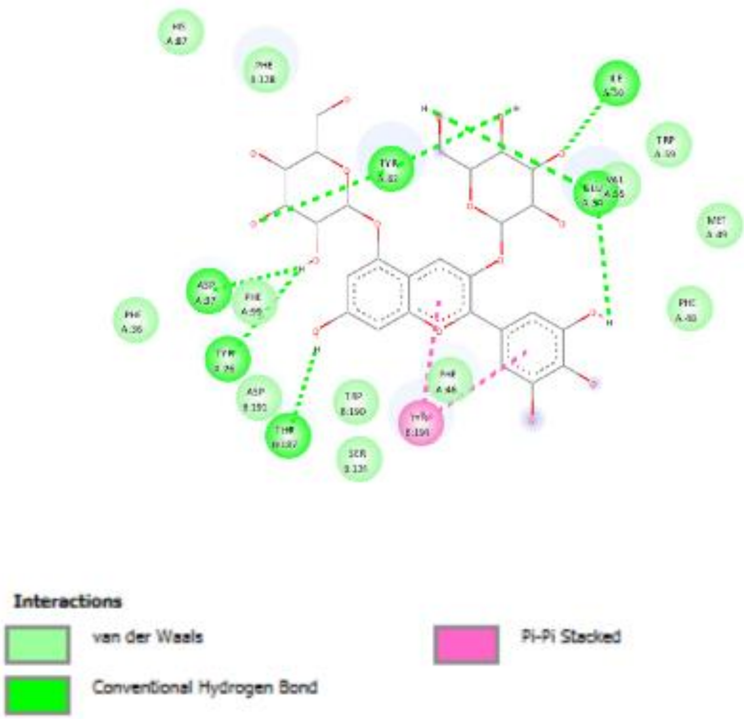

4	Naringin	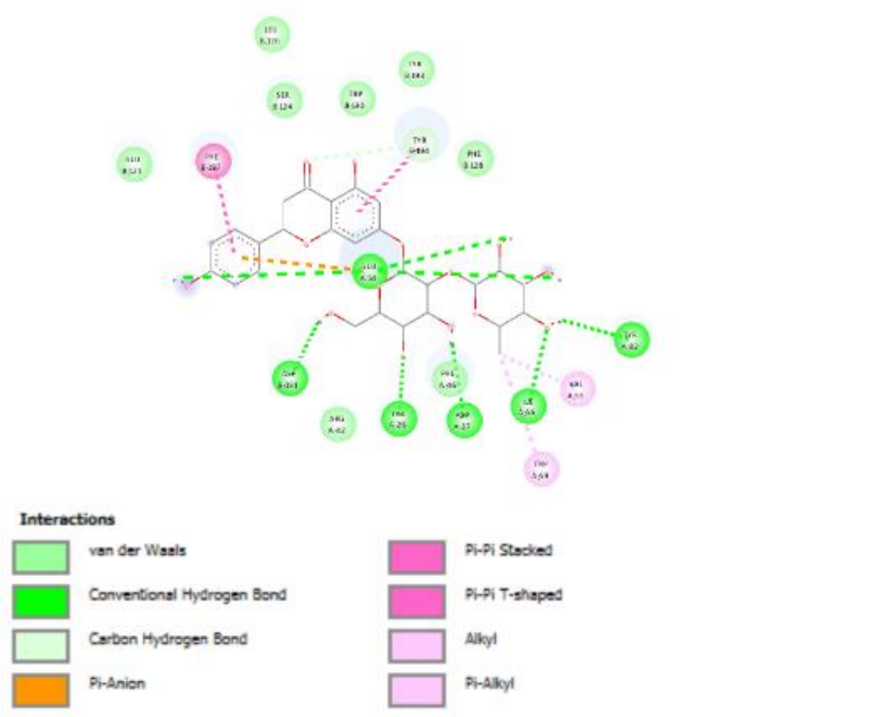Interactions<br>van der Waals<br>Conventional Hydrogen Bond<br>Carbon Hydrogen Bond<br>Pi-Anion<br>Pi-Pi Stacked<br>Pi-Pi T-shaped<br>Alkyl<br>Pi-Alkyl

5	Rutin	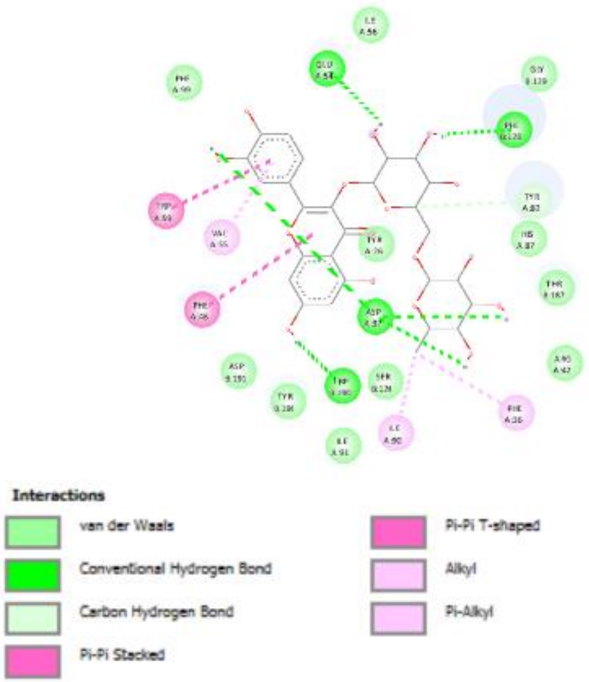Interactions<br>van der Waals<br>Conventional Hydrogen Bond<br>Carbon Hydrogen Bond<br>Pi-Pi Stacked<br>Pi-Pi T-shaped<br>Alkyl<br>Pi-Alkyl

6	Shikimic Acid	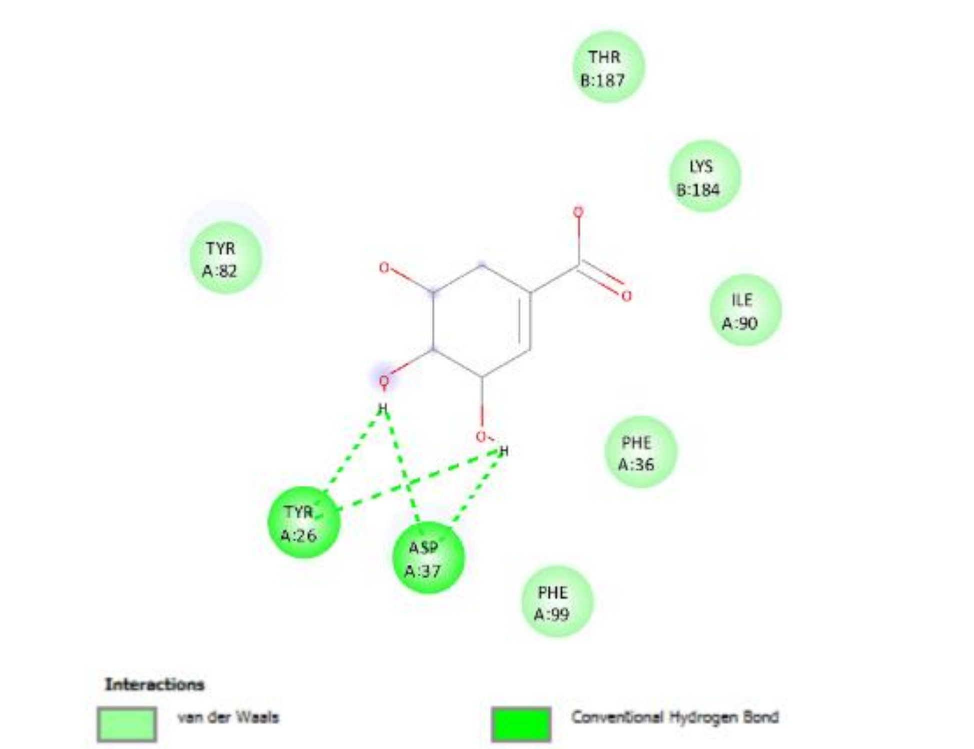

ROS1 Kinase

No.	Substance	Visualization
Native Ligand		

1	3ZBF	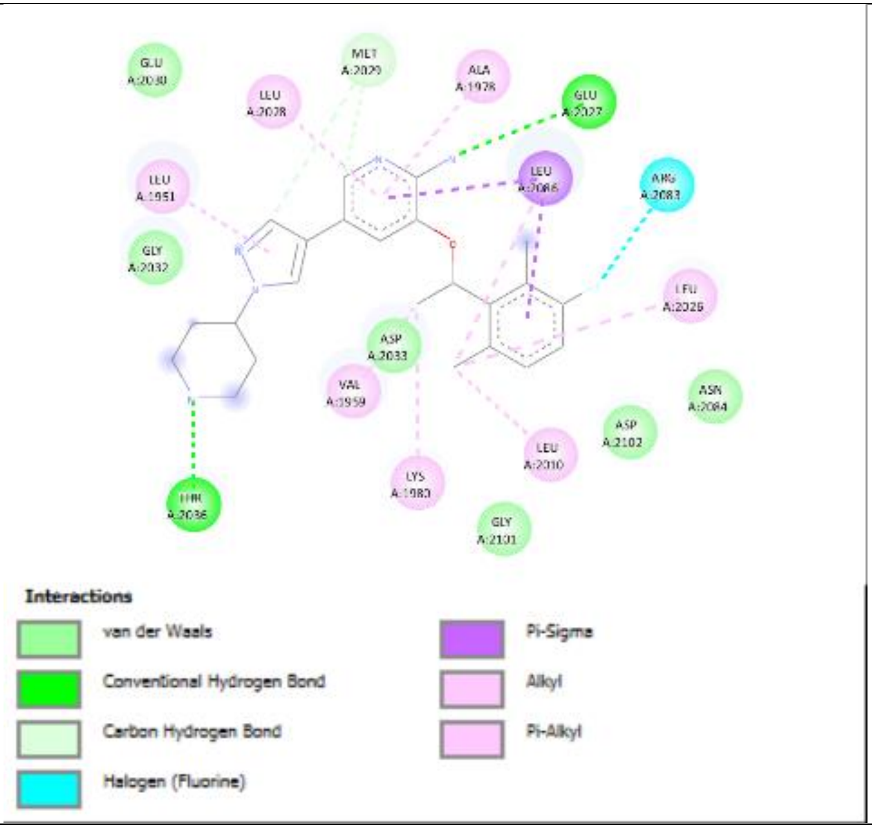	
Control			

<table>
<tr><td>1</td><td>Luteolin</td><td>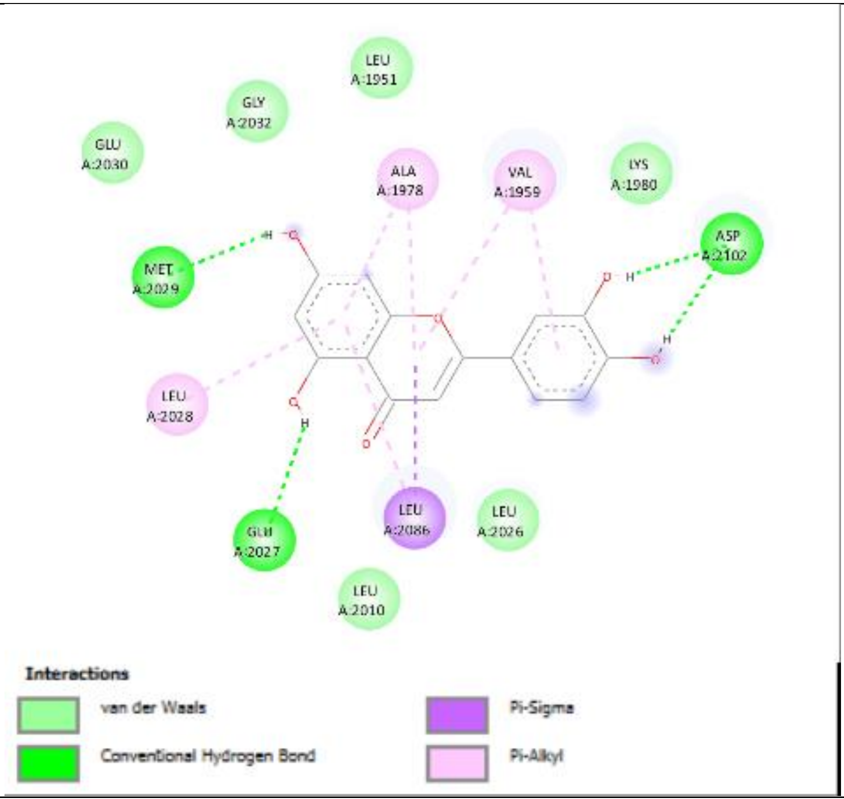
</td><td></td></tr>
<tr><td colspan="2">aCSE</td><td colspan="2"></td></tr>
</table>

1	Epicatechin	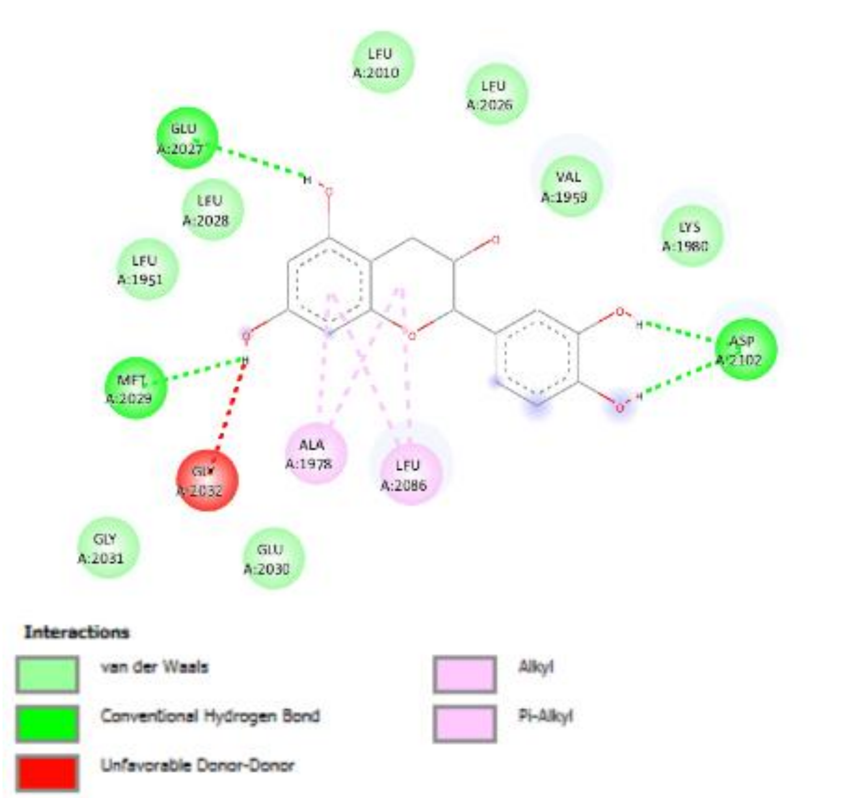

2	Gallic acid	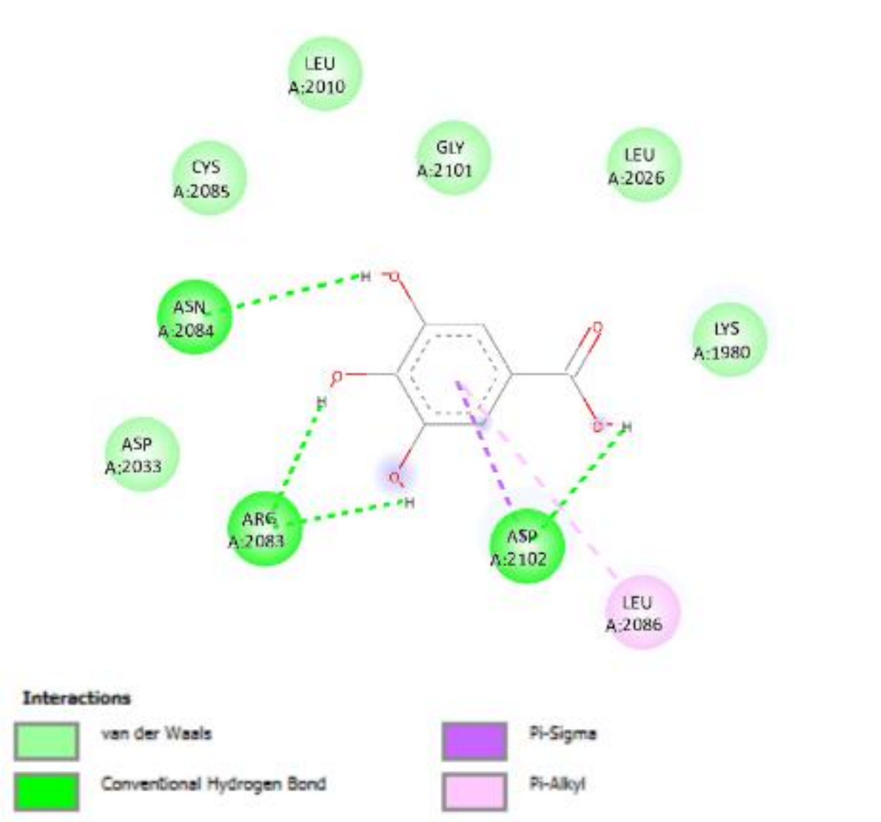

3	4-Hydroxycinnamic acid	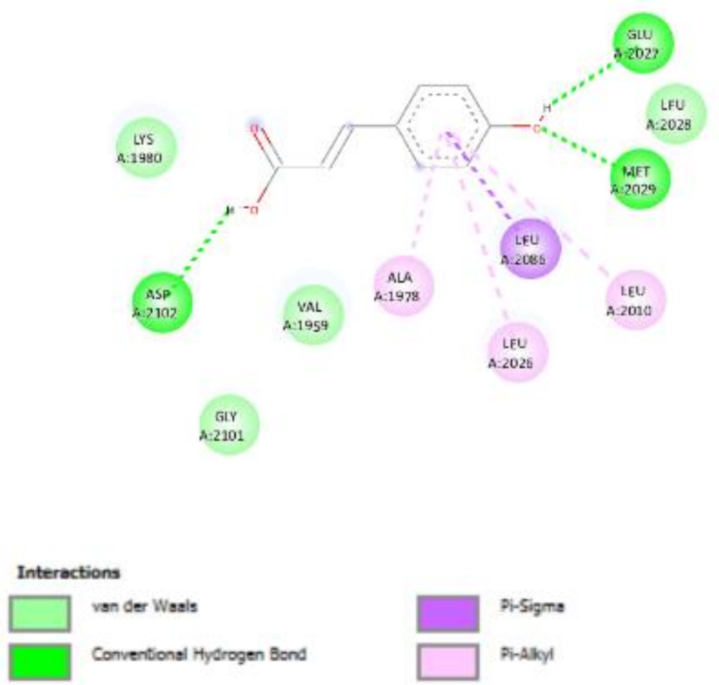

4	Kaempferol	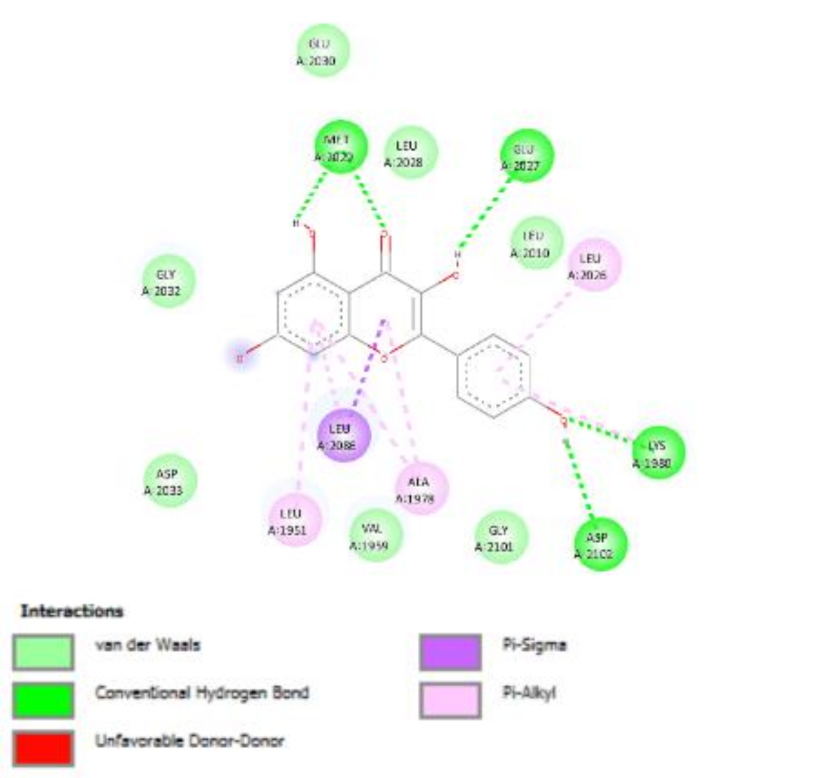

5	Quercitrin	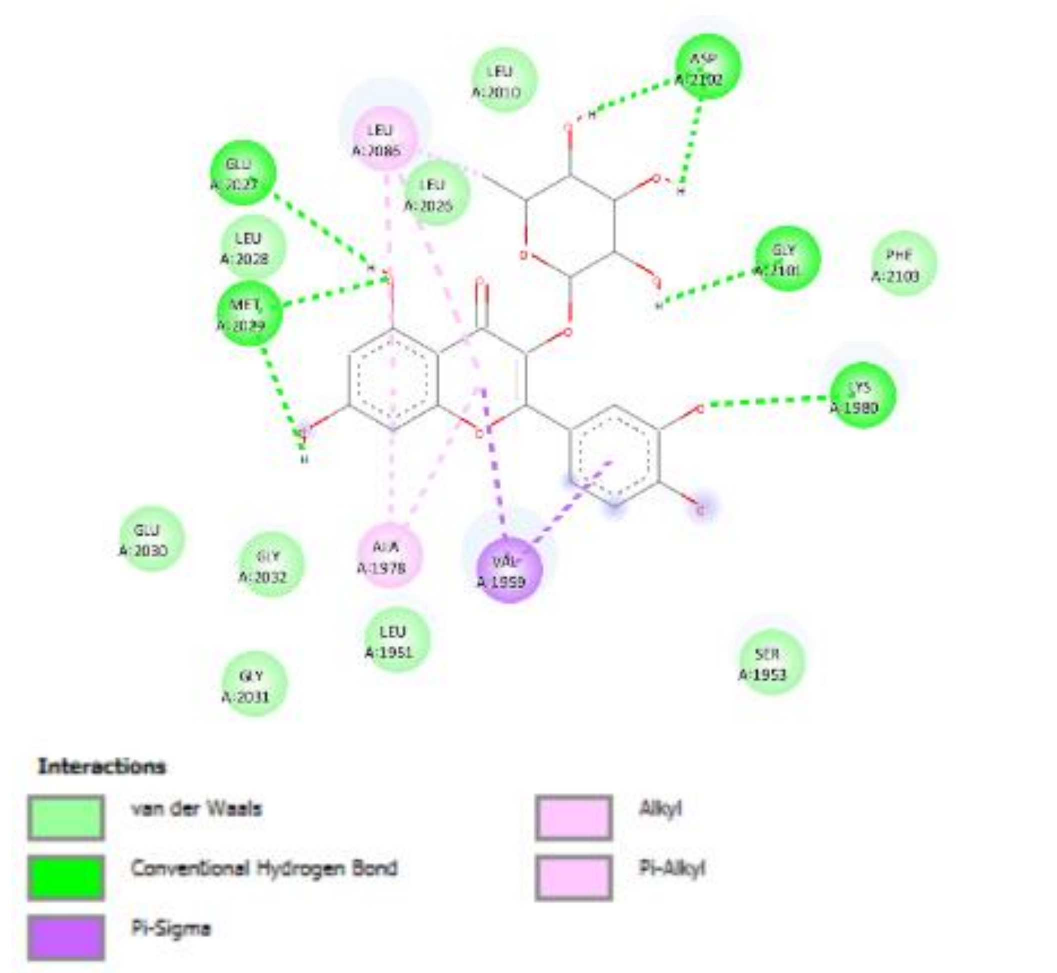
rCSE		

1	Caffeic acid	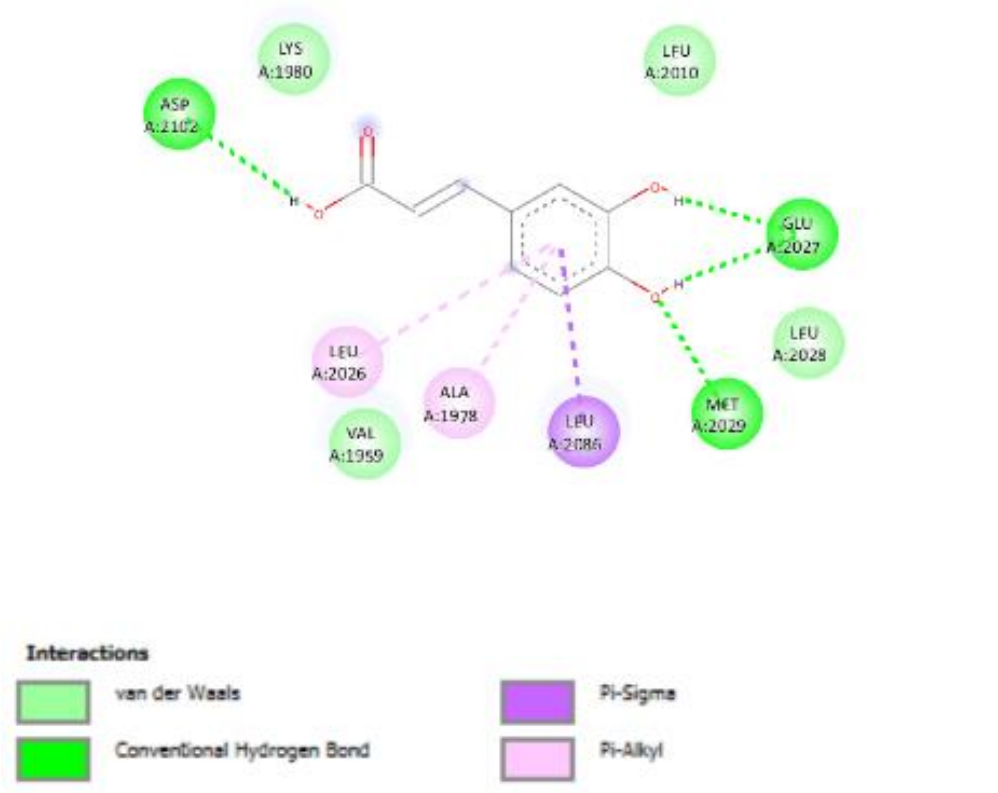

2	(+)-Catechin	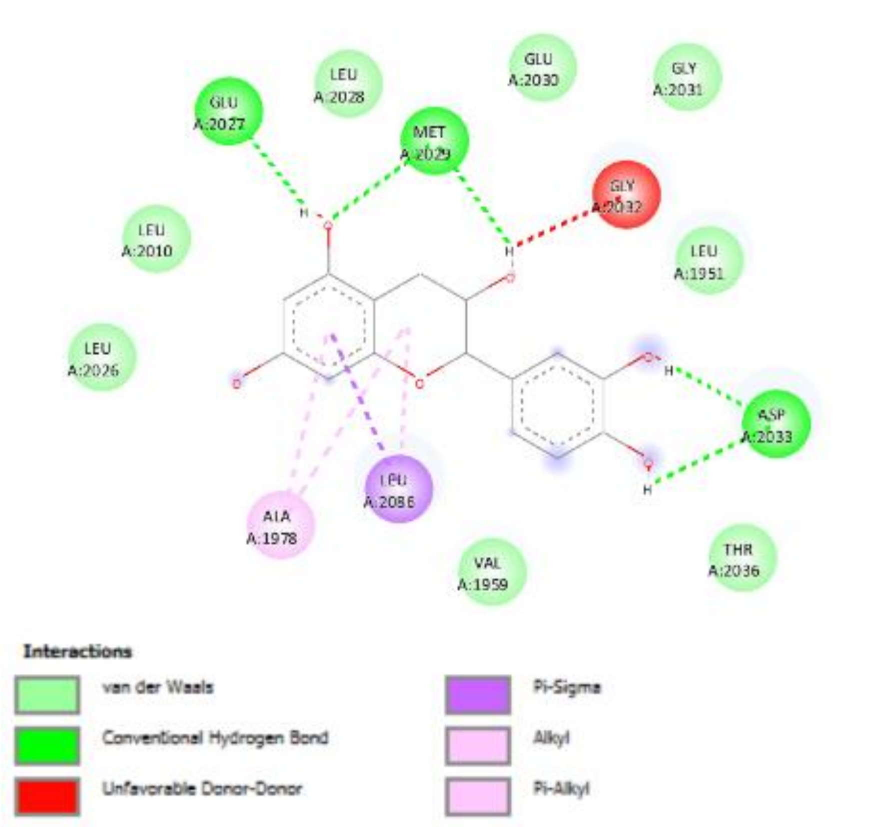

GLU A:2027
LEU A:2028
GLU A:2030
GLY A:2031
MET A:2029
GLY A:2032
LEU A:2010
LEU A:1951
LEU A:2026
ASP A:2033
LEU A:2086
ALA A:1978
VAL A:1959
THR A:2036
Interactions
van der Waals
Conventional Hydrogen Bond
Unfavorable Donor-Donor
Pi-Sigma
Alkyl
Pi-Alkyl

3	(2R,3S,4S,5R,6S)-2-(hydroxymethyl)-6-[7-hydroxy-3-[(2S,3R,4S,5S,6R)-3,4,5-trihydroxy-6-(hydroxymethyl)oxan-2-yl]oxy-2-(3,4,5-trihydroxyphenyl)chromenylium-5-yl]oxyoxane-3,4,5-triol	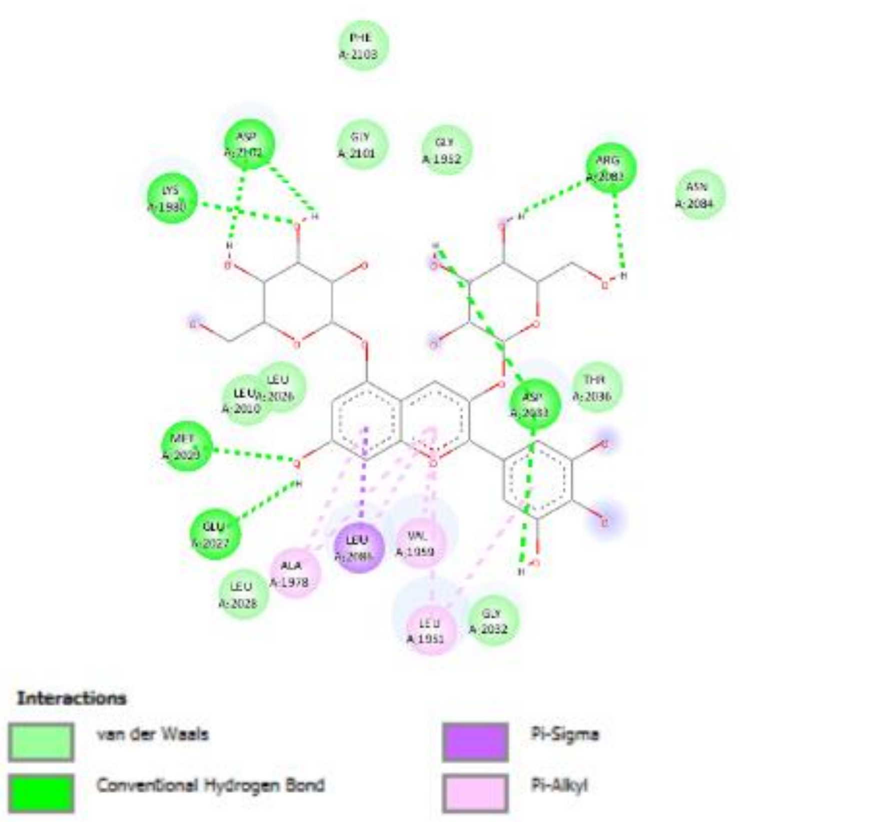

4	Naringin	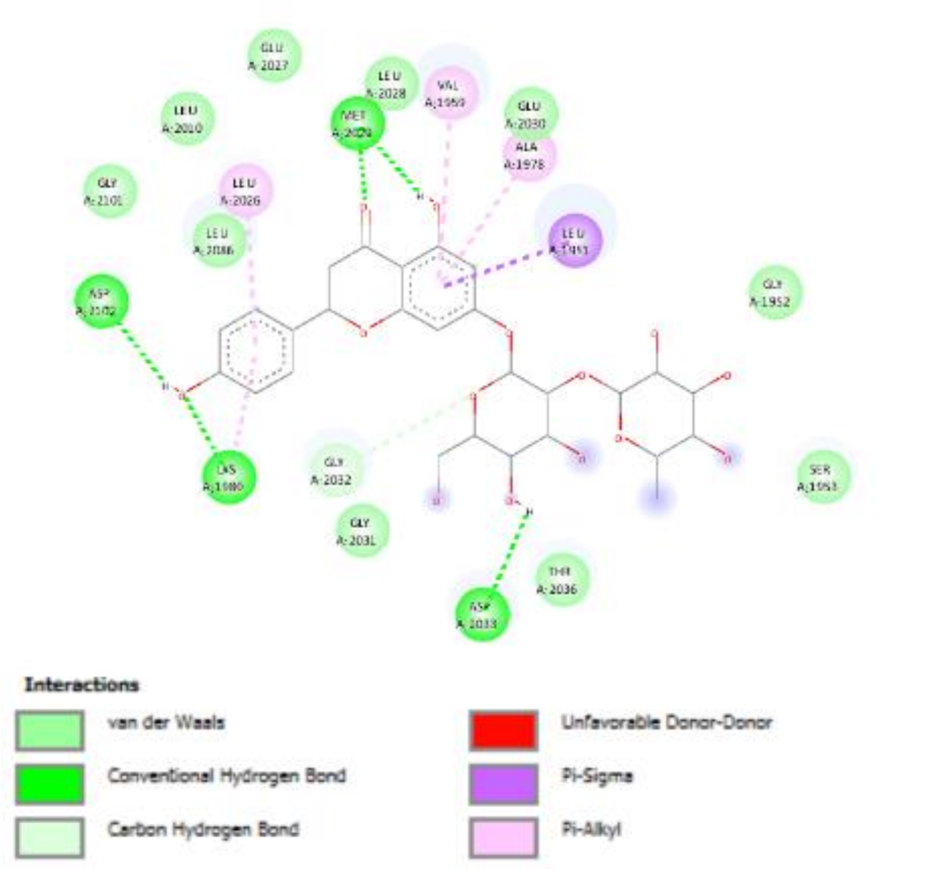GLU A:2027<br>LEU A:2028<br>GLU A:2030<br>ALA A:1978<br>LEU A:2010<br>GLY A:2101<br>LEU A:2026<br>GLY A:1952<br>GLY A:2032<br>SER A:1953<br>GLY A:2031<br>THR A:2036<br>Interactions<br>van der Waals<br>Conventional Hydrogen Bond<br>Carbon Hydrogen Bond<br>Unfavorable Donor-Donor<br>Pi-Sigma<br>Pi-Alkyl

5	Rutin	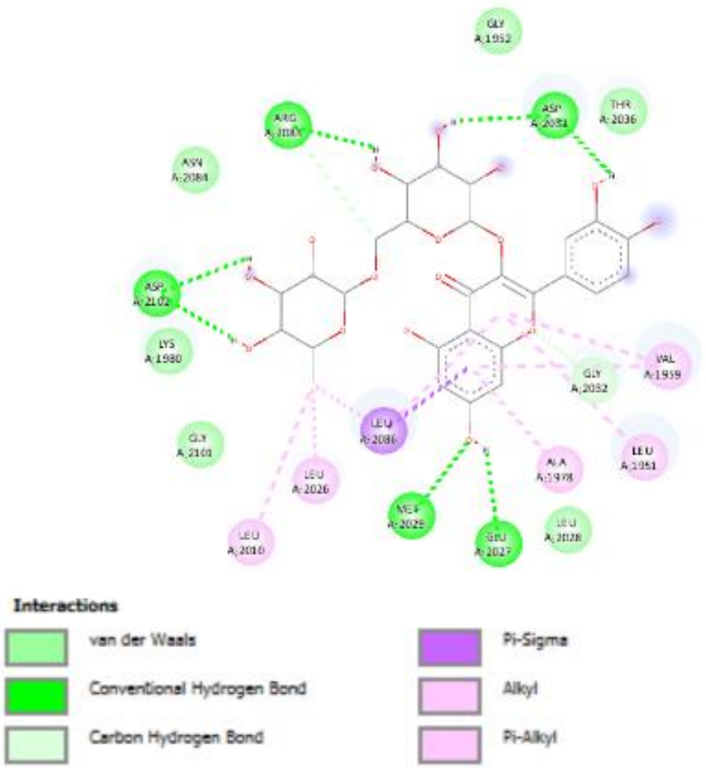 Interactions<br>van der Waals<br>Conventional Hydrogen Bond<br>Carbon Hydrogen Bond<br>Pi-Sigma<br>Alkyl<br>Pi-Alkyl

6	Shikimic Acid	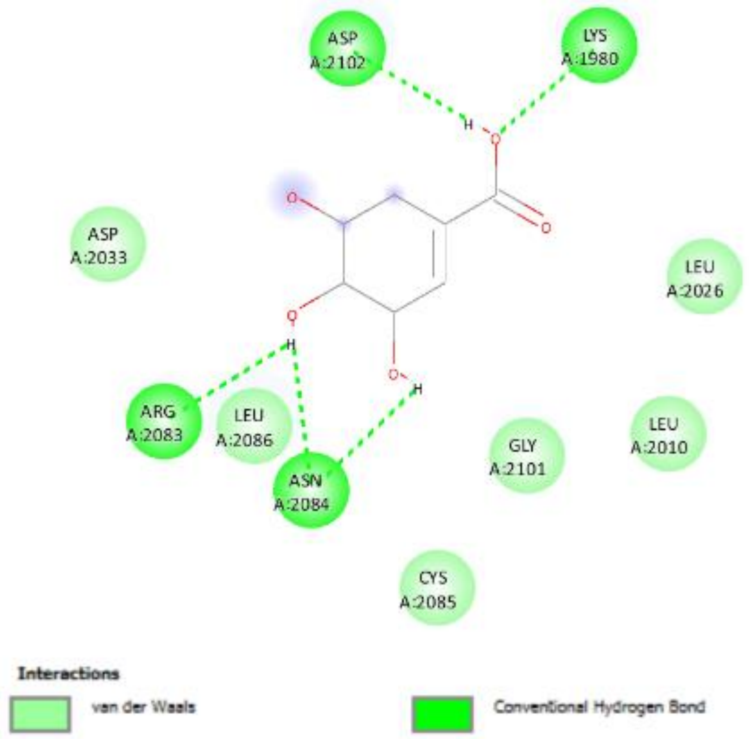

HIF-1alpha

No.	Substance	Visualization
Native Ligand		

1	1H2N	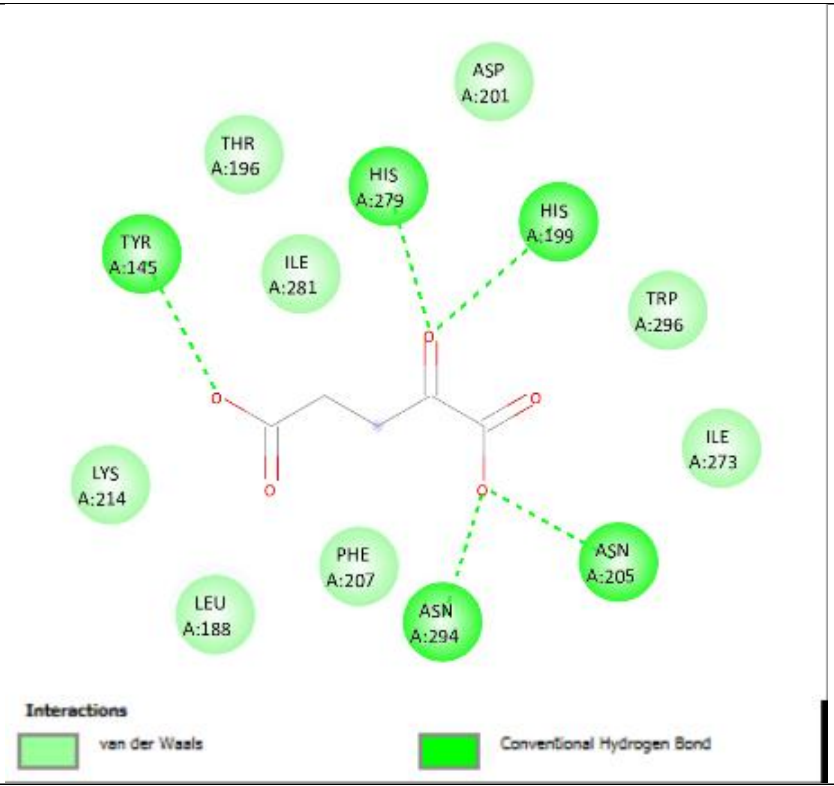
Control		

<table>
<tr><td>1</td><td>Genistein</td><td>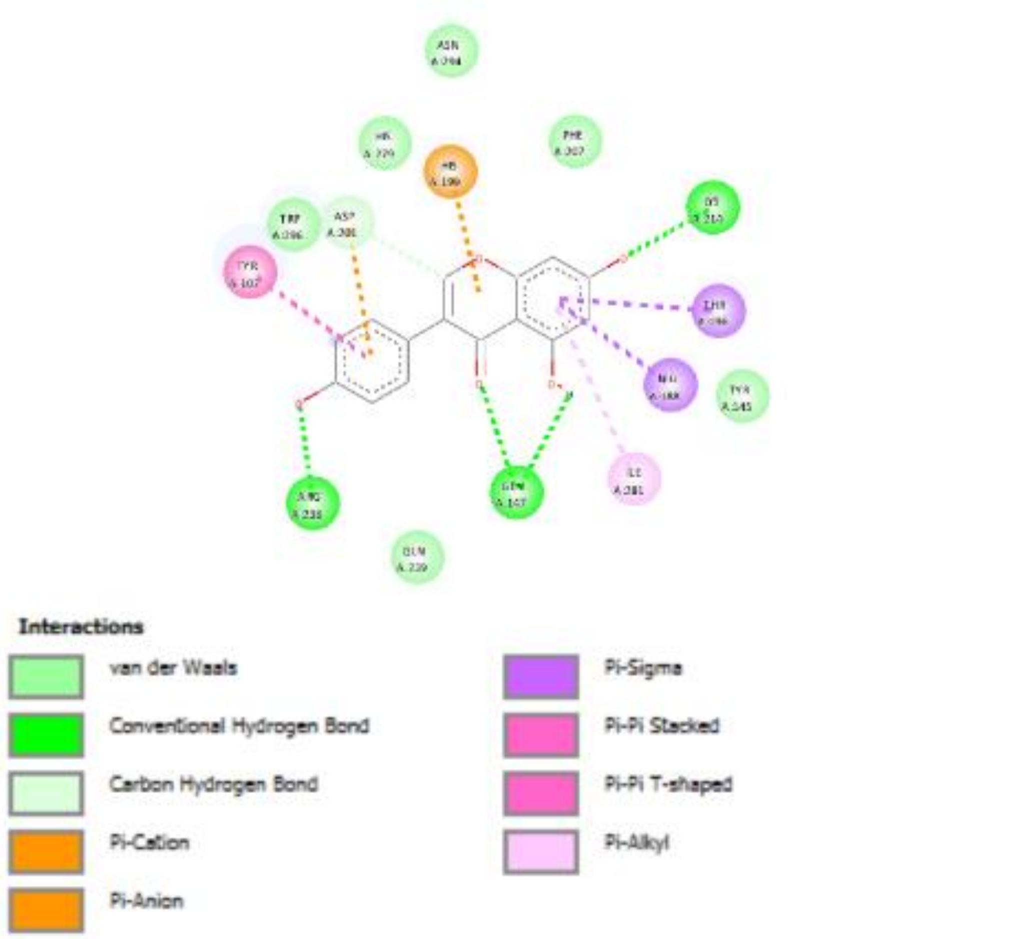
</td></tr>
<tr><td colspan="2">aCSE</td><td></td></tr>
</table>

1	Epicatechin	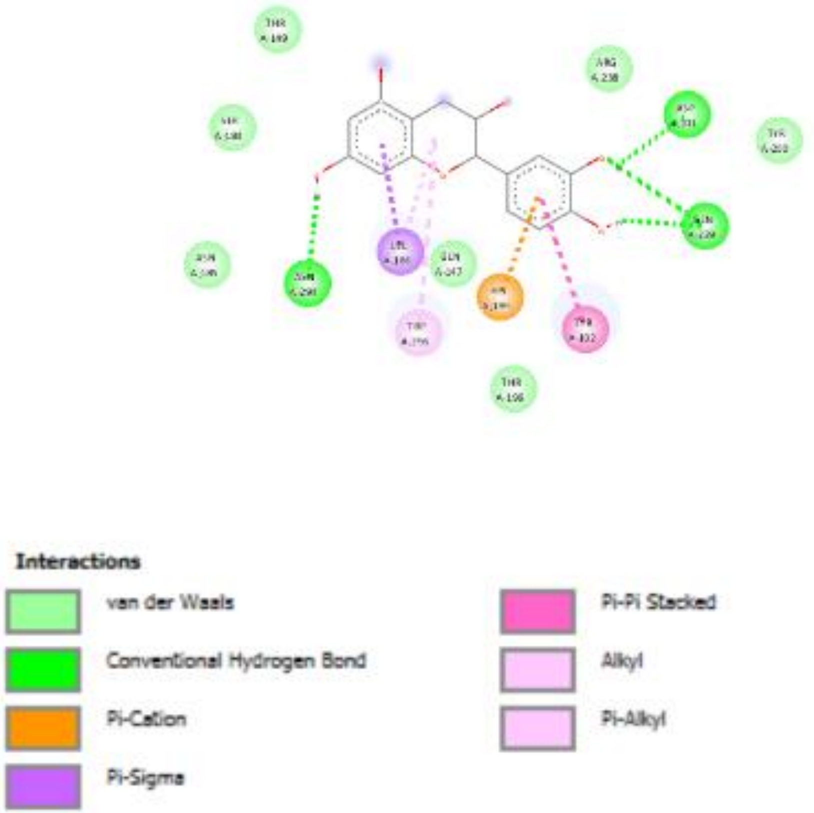

2	Gallic acid	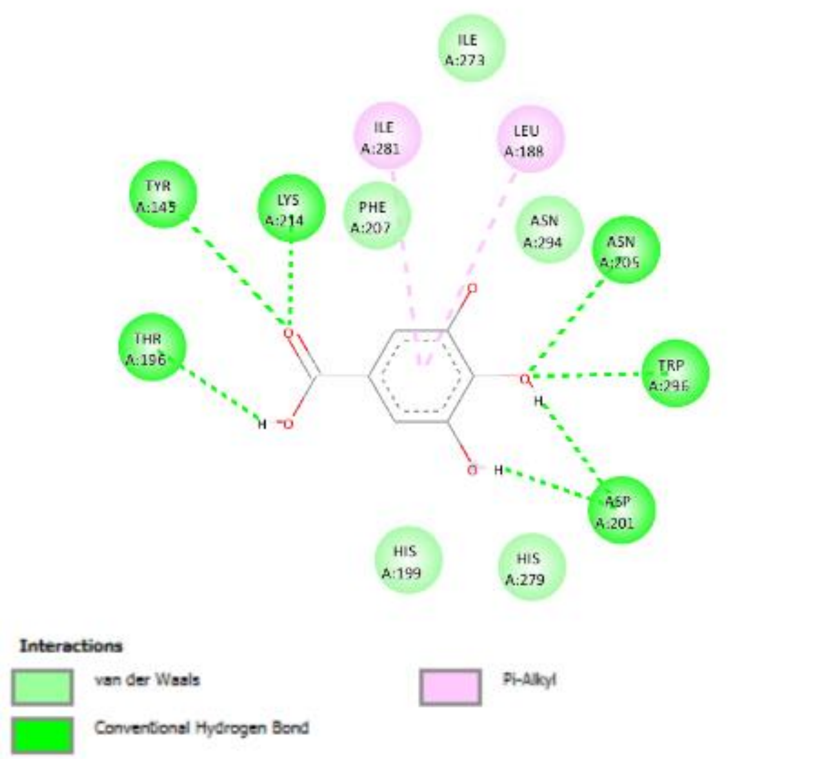

3	4-Hydroxycinnamic acid	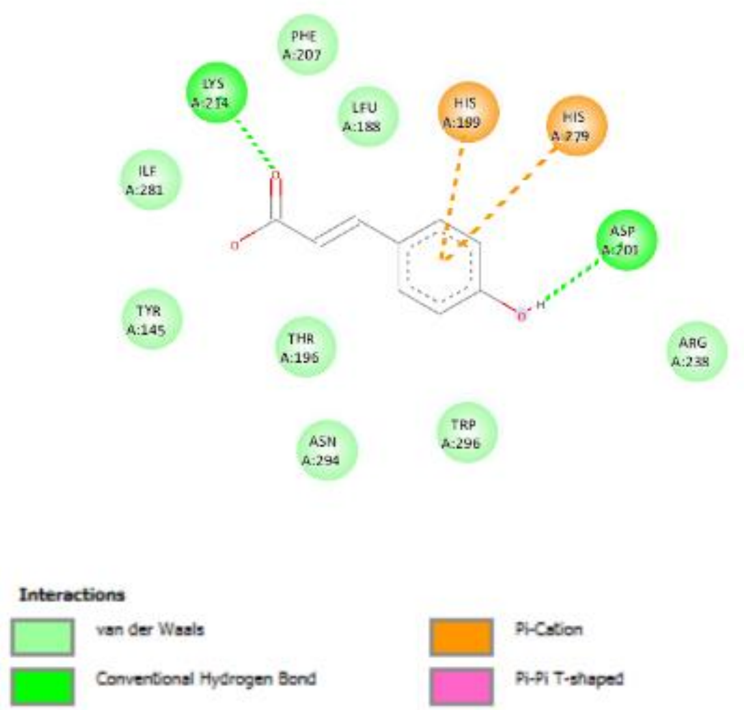

4	Kaempferol	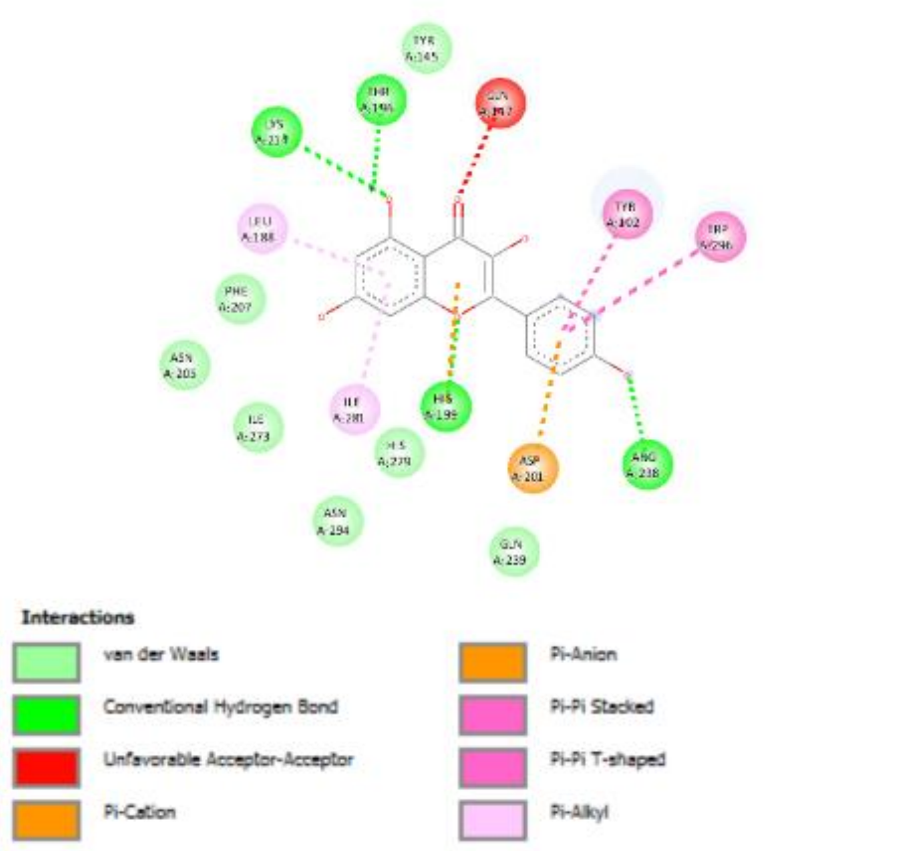

5	Quercitrin	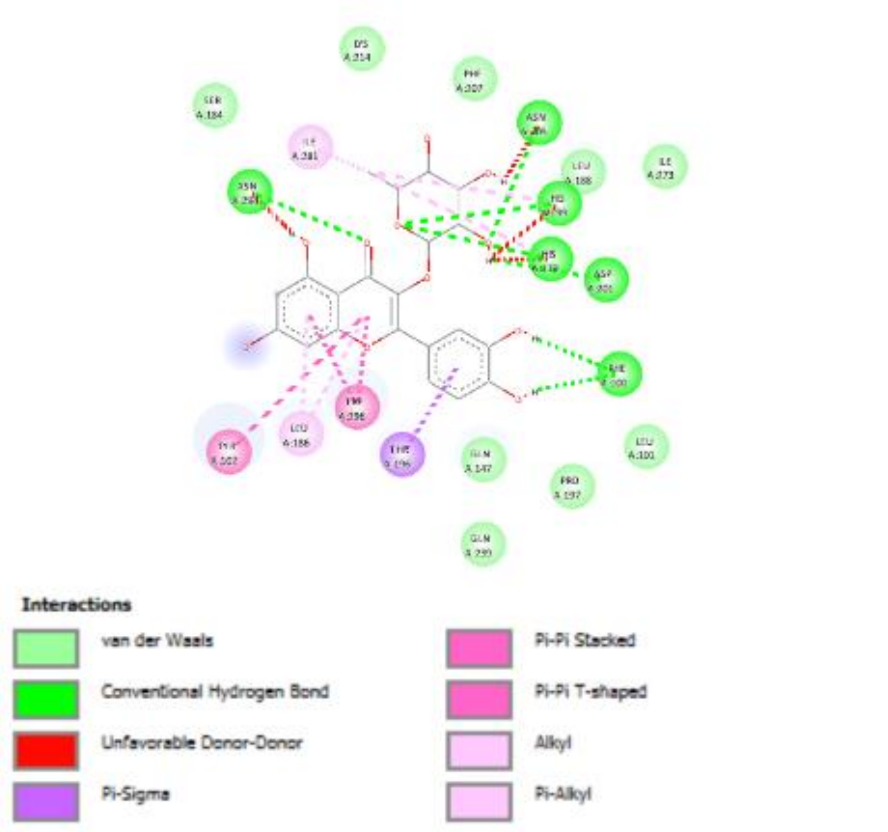
rCSE		

1	Caffeic acid	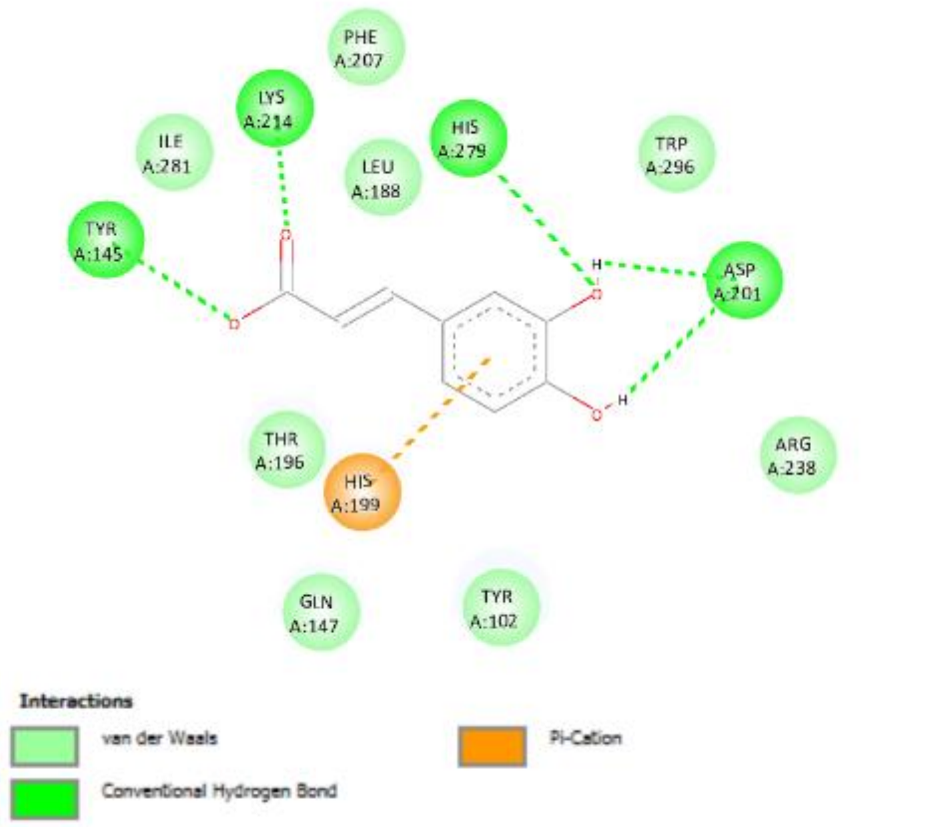

2	(+)-Catechin	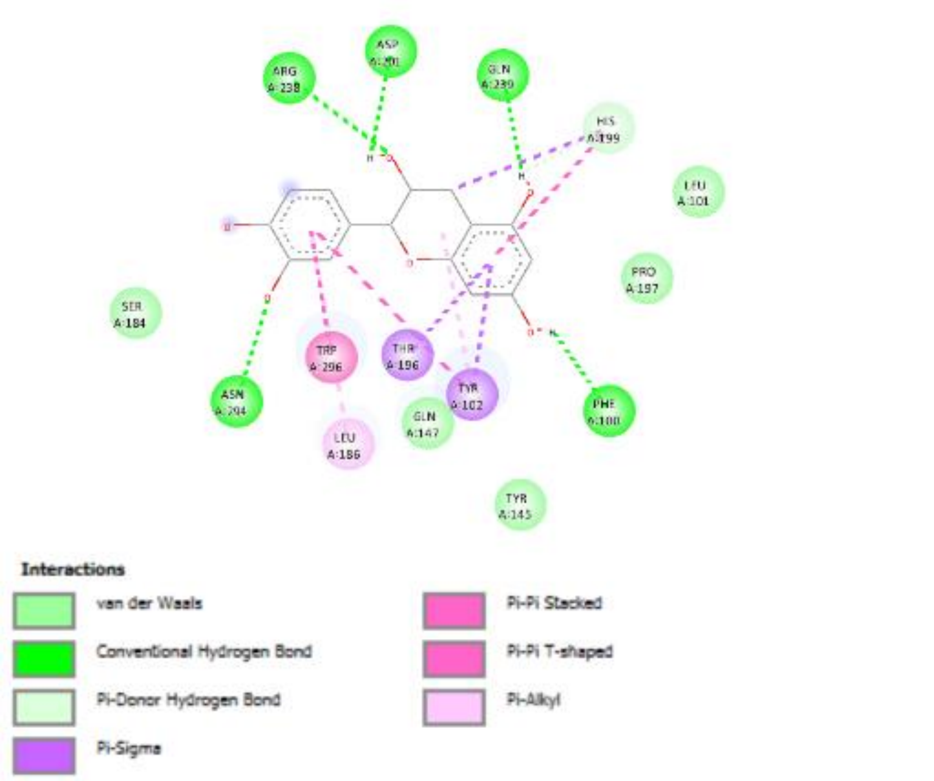

ARG A:238
GLN A:239
HIS A:199
LEU A:101
PRO A:197
SER A:184
TRP A:296
THR A:196
TYR A:102
GLN A:147
PHE A:100
LEU A:186
TYR A:145
Interactions
van der Waals
Conventional Hydrogen Bond
Pi-Donor Hydrogen Bond
Pi-Sigma
Pi-Pi Stacked
Pi-Pi T-shaped
Pi-Alkyl

3	(2R,3S,4S,5R,6S)-2-(hydroxymethyl)-6-[7-hydroxy-3-[(2S,3R,4S,5S,6R)-3,4,5-trihydroxy-6-(hydroxymethyl)oxan-2-yl]oxy-2-(3,4,5-trihydroxyphenyl)chromenylium-5-yl]oxyoxane-3,4,5-triol	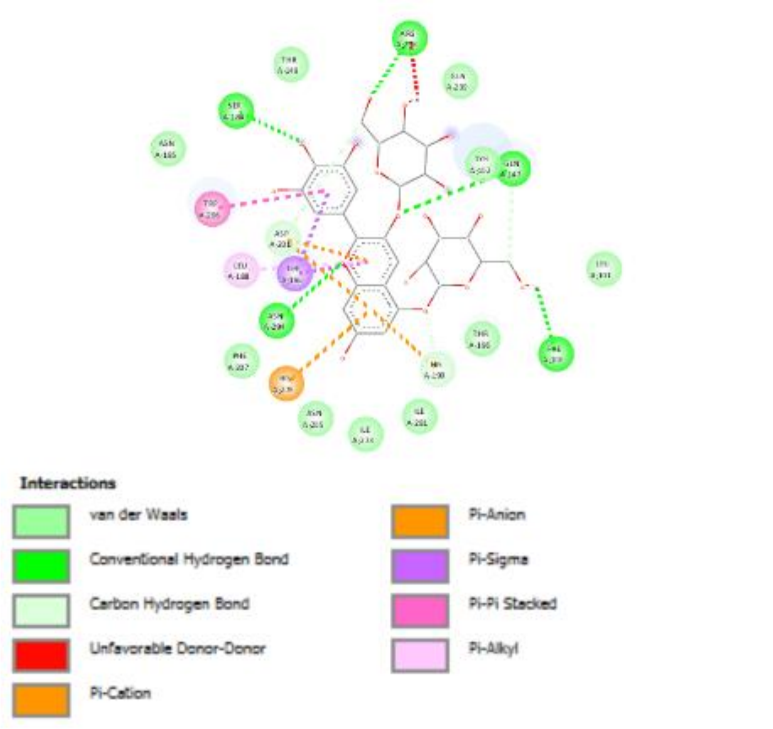

4	Naringin	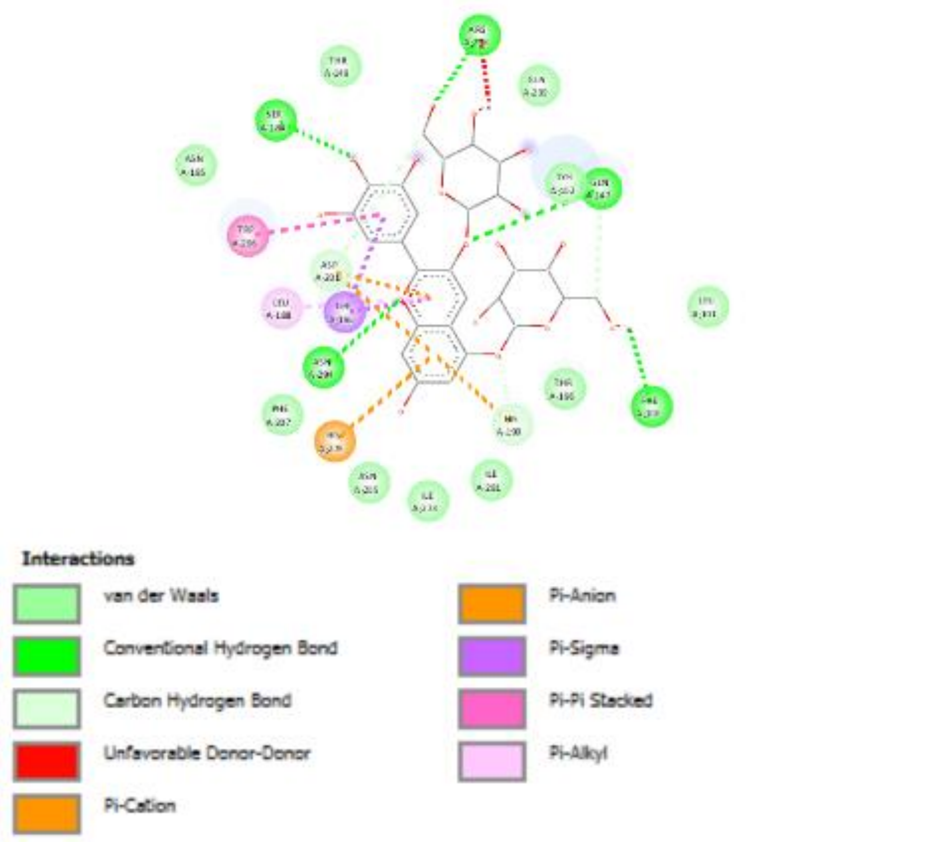 Interactions<br>van der Waals<br>Conventional Hydrogen Bond<br>Carbon Hydrogen Bond<br>Unfavorable Donor-Donor<br>Pi-Cation<br>Pi-Anion<br>Pi-Sigma<br>Pi-Pi Stacked<br>Pi-Alkyl

5	Rutin	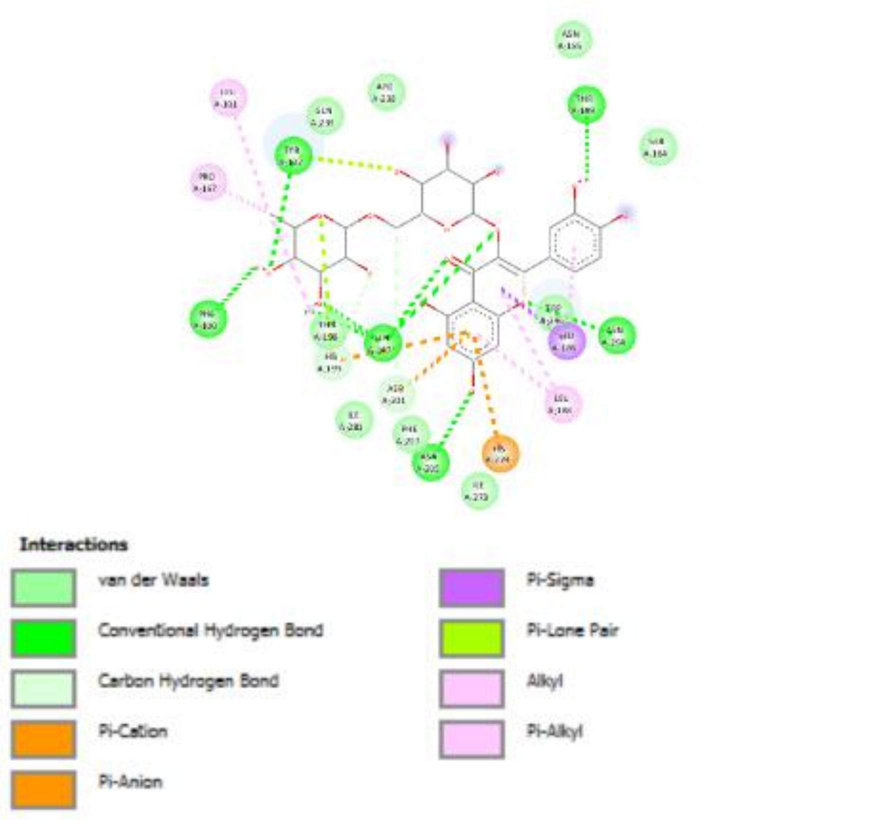Interactions<br>van der Waals<br>Conventional Hydrogen Bond<br>Carbon Hydrogen Bond<br>Pi-Cation<br>Pi-Anion<br>Pi-Sigma<br>Pi-Lone Pair<br>Alkyl<br>Pi-Alkyl

6	Shikimic Acid	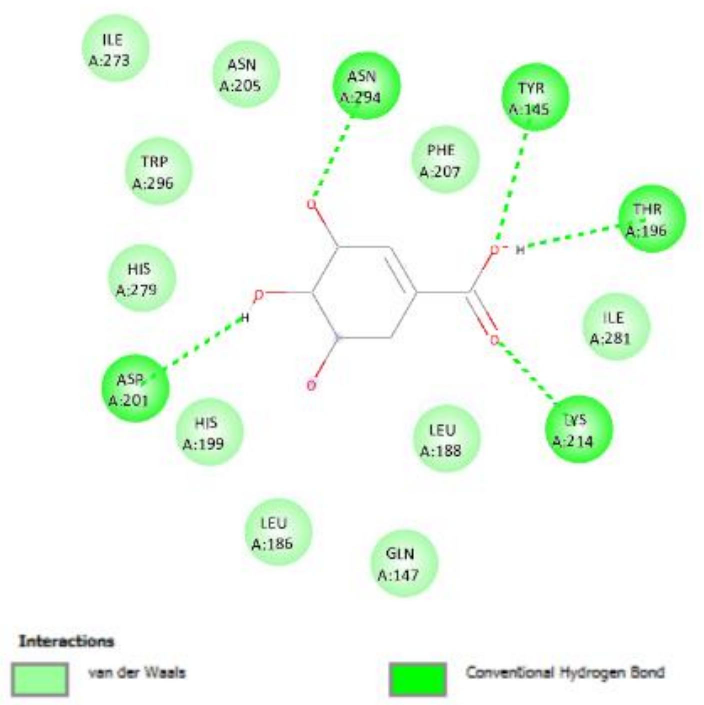
